# Characterization of a Triplet Vinylidene

**DOI:** 10.1021/jacs.1c11062

**Published:** 2021-12-13

**Authors:** Yury Kutin, Justus Reitz, Patrick W. Antoni, Anton Savitsky, Dimitrios A. Pantazis, Müge Kasanmascheff, Max M. Hansmann

**Affiliations:** † Department of Chemistry and Chemical Biology, Technische Universität Dortmund, Otto-Hahn-Str. 6, 44227 Dortmund, Germany; ‡ Department of Physics, Technische Universität Dortmund, Otto-Hahn-Str. 4a, 44227 Dortmund, Germany; § Max-Planck-Institut für Kohlenforschung, Kaiser-Wilhelm-Platz 1, 45470 Mülheim an der Ruhr, Germany

## Abstract

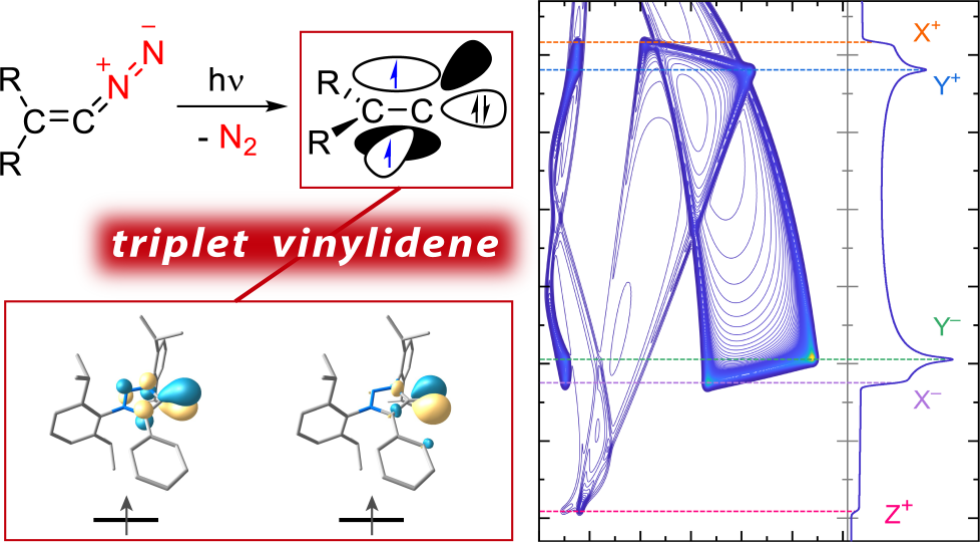

Singlet vinylidenes (R_2_C=C:) are proposed as intermediates in a series of organic reactions, and very few have been studied by matrix isolation or gas-phase spectroscopy. Triplet vinylidenes, however, featuring two unpaired electrons at a monosubstituted carbon atom are thus far only predicted as electronically excited-state species and represent an unexplored class of carbon-centered diradicals. We report the photochemical generation and low-temperature EPR/ENDOR characterization of the first ground-state high-spin (triplet) vinylidene. The zero-field splitting parameters (*D* = 0.377 cm^–1^ and |*E|*/*D* = 0.028) were determined, and the ^13^C hyperfine coupling tensor was obtained by ^13^C-ENDOR measurements. Most strikingly, the isotropic ^13^C hyperfine coupling constant (50 MHz) is far smaller than the characteristic values of triplet carbenes, demonstrating a unique electronic structure which is supported by quantum chemical calculations.

## Introduction

**Figure 1:**
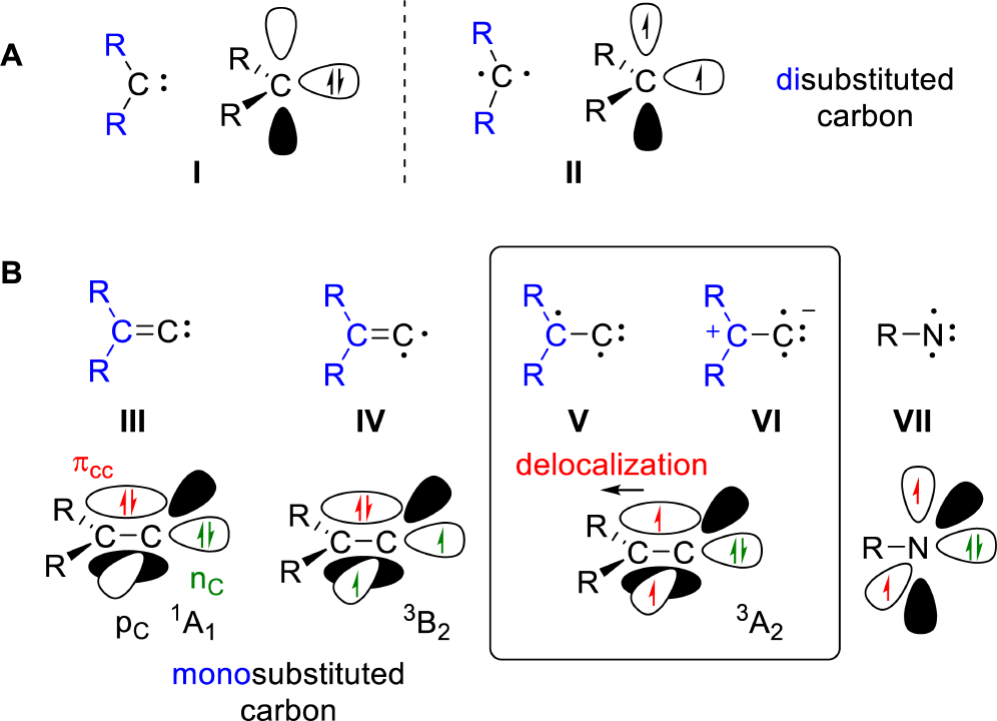
Fundamental compound classes. (A) Comparison between singlet and triplet carbenes (**I**/**II**). (B) Singlet (**III**) and triplet vinylidenes (**IV–VI**) as well as triplet nitrenes (**VII**). The simplified depiction of electronic structure does not imply that all relevant states are necessarily of single-reference character.

The synthesis of paramagnetic organic compounds such as radicals or diradicals has fascinated chemists since Gomberg’s seminal discovery of the stable triphenylmethyl radical in 1900.^1^ In particular, high-spin ground-state species such as diradicals are typically challenging to study but are highly attractive for a series of applications due to their magnetic properties.^2−4^ The isolation and characterization of the first stable divalent carbon compounds (R_2_C:), i.e., singlet carbenes (**I**) by Bertrand^5^ and Arduengo^6^ as well as persistent triplet carbenes (**II**) by Tomioka,^7^ represented a breakthrough and paradigm shift for chemistry (Figure 1A).^8^

The higher homologues, unsaturated carbenes or vinylidenes (Figure 1B) in which carbon is bound to only one substituent, have been much less explored.^9^ Besides the well-known metal vinylidenes,^10^ free vinylidenes (**III**) have been postulated to be involved in a series of important organic reactions such as the Seyferth-Gilbert homologation.^11^ Besides computational data,^12,13^ very few data on singlet vinylidenes exist, which are based on matrix isolation^14,15^ or gas phase spectroscopy.^16−18^ Singlet H_2_C=C: has been proposed in combustion processes and rearranges on the picosecond time scale to acetylene.^19^ In addition to the singlet state, vinylidenes also feature two symmetry-distinct triplet states of similar energy, which are typically high-lying electronically excited states.^20^ The promotion of an electron from the spin-paired σ(sp) orbital to the empty p orbital (n_C_ → p_C_) leads to **IV** (^3^B_2_) (Figure 1). Promoting an electron from the C=C π orbital to the vacant p orbital (π_CC_ → p_C_) results in triplet **V**/**VI** (^3^A_2_). Interestingly, the lowest metastable electronically excited state of H_2_C=C [Δ(^1^A_1_ – ^3^B_2_) ≈ 40 kcal/mol] rearranges to acetylene much more slowly than does singlet vinylidene.^21−23^ Note that R_2_C^+^–C^–^ (**VI**) is isoelectronic to (cationic) triplet nitrenes (**VII**), which feature a triplet ground state with two singly occupied orthogonal p orbitals (^3^A_2_).^24^ It is well known that the delocalization of unpaired electrons in triplet carbenes (or nitrenes) into adjacent π systems leads to increased stability.^25^ Since **V/VI** features only one substituent, we envisioned the stabilization of a triplet vinylidene by the delocalization of one electron into an adjacent heterocycle, while the second electron remains centered on carbon. Importantly, destabilizing the singlet state by disfavoring a C=C double bond should be possible by starting from ylidic polarized/mesoionic systems.^26^

To the best of our knowledge, there are no experimental data on any ground-state triplet vinylidene. In contrast to the rich chemistry of triplet carbenes,^27^ triplet vinylidenes remain unexplored. One obvious reason might be the missing synthetic access, which was highlighted by Stang in 1978, suggesting diazoalkenes (R_2_C=C=N_2_) to be the best vinylidene precursors.^28^ However, stable diazoalkenes were unknown until we reported our recent synthesis of the first room-temperature-stable representative,^29,30^ which now enables the entry point into this fundamental compound class. Here, we report the photogeneration and characterization of a ground-state triplet vinylidene using electron paramagnetic resonance (EPR) spectroscopy.

## Results and Discussion

**Figure 2:**
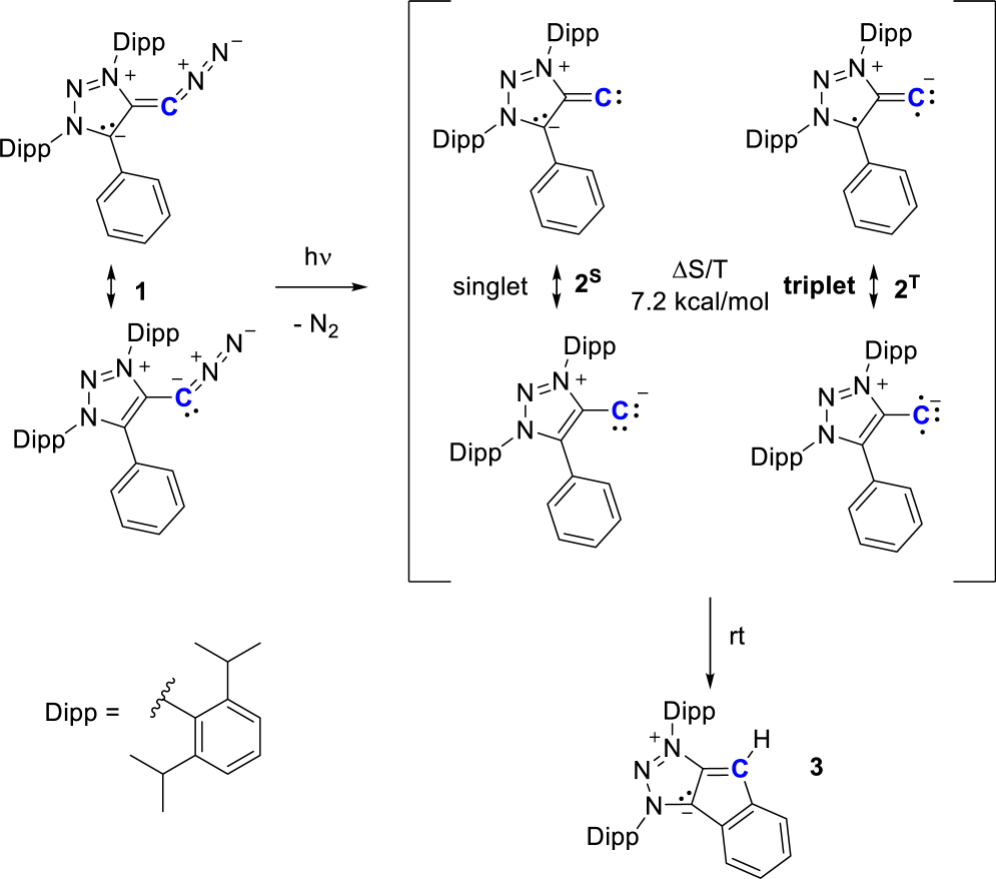
Irradiation of diazoalkene **1** leads to C–H insertion product **3** via ground-state triplet vinylidene **2**. ^13^C labeling is shown in blue.

Irradiating a benzene solution of diazoalkene **1**(31) with a 390 nm LED for 30 min at room temperature cleanly generates C–H insertion product **3**, as evidenced by *in situ*^1^H NMR spectroscopy (Figure 2 and Figure S1). Mechanistically, the photochemically triggered loss of N_2_ should lead to vinylidene **2** in either a singlet (**2**^**S**^) or triplet (**2**^**T**^) ground state. Hybrid density functional theory B3LYP/def2-TZVP calculations on the geometry-optimized structures of **2** favor the triplet state^32^ by 7.2 kcal/mol. Additionally, calculations with the benchmark-quality^33^ DLPNO-CCSD(T1) coupled-cluster approach on a simplified model of **2** where ring substituent groups were replaced by hydrogens suggest a singlet–triplet gap of 13.1 kcal/mol. This result is corroborated by multireference perturbation theory NEVPT2 calculations that use a full-valence active space of 10 electrons in 8 orbitals, which locate a closed-shell singlet state at 12.2 kcal/mol and an open-shell singlet state at 13.6 kcal/mol above the triplet ground state. (See the SI for the detailed methodology and results.) These results uniformly support the remarkable assignment of a spin-triplet ground state for vinylidene **2**, which is well separated from excited spin-singlet states.

**Figure 3:**
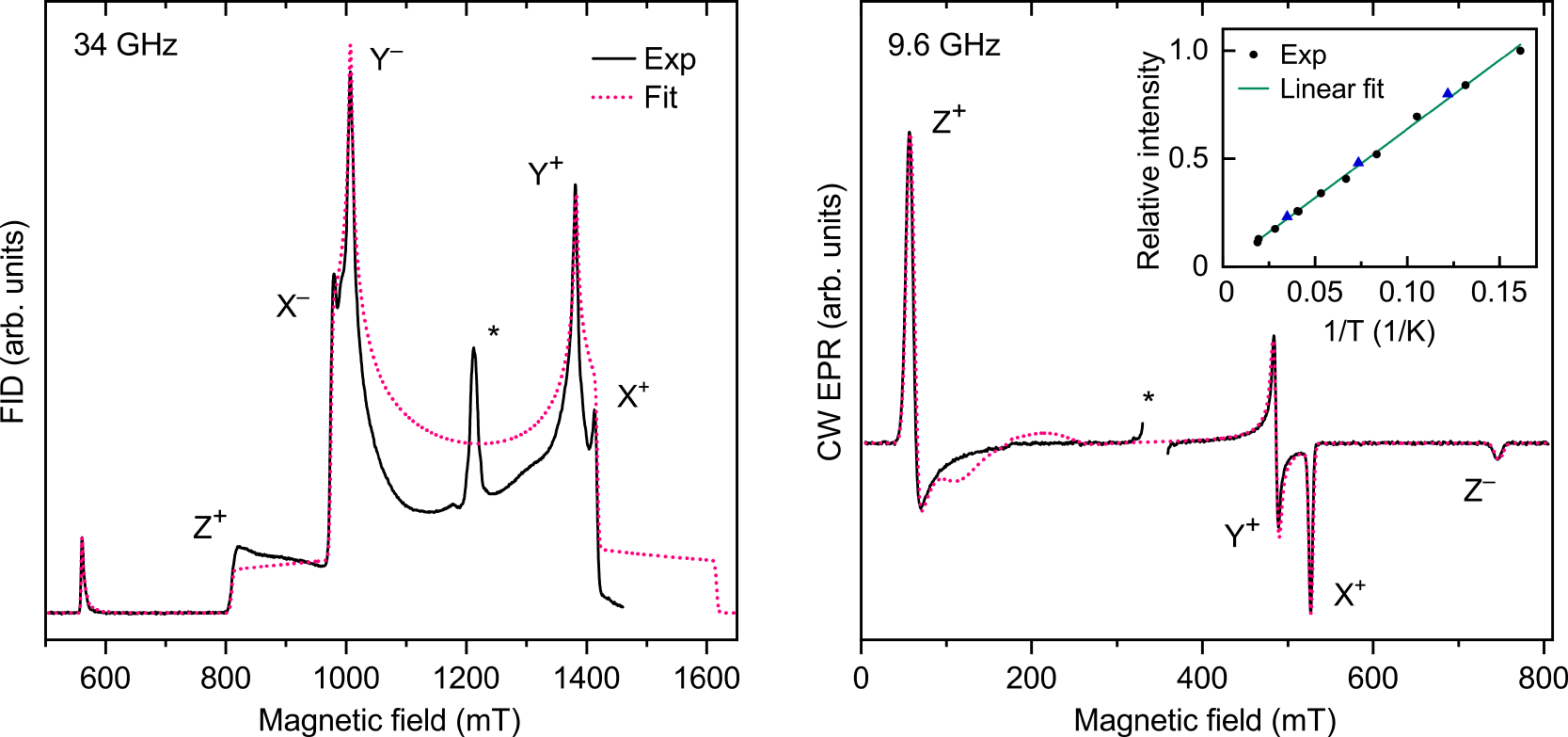
(Left) FID-detected Q-band EPR spectrum (black solid line) acquired at 6 K after the photolysis of **1** (Hg arc lamp, 1 h at 10 K) in frozen toluene solution (20 mM). The spectrum is overlaid with the best fit achieved assuming an *S* = 1 species (magenta dotted line). Canonical orientations of the ZFS tensor are labeled with *X*, *Y*, and *Z*. The superscripts refer to the *M*_S_ = −1 ↔ 0 (−) and *M*_S_ = 0 ↔ 1 (+) transitions. The intensity of the simulated half-field (*M*_S_ = −1 ↔ 1) signal at 560 mT was reduced by 86% to account for a difference in transition probabilities. The narrow signal marked with an asterisk originates from a minor fraction of photolysis byproducts (radicals and radical pairs) typically observed for divalent carbenes.^40^ (Right) CW X-band EPR spectrum acquired at 7 K after the photolysis of **1** (Xe arc lamp, 1 h at 7 K) in frozen toluene solution (6 mM), overlaid with the best fit. The radical signal is omitted for clarity. (Inset) CW EPR intensity vs inverse temperature. (Black circles) Temperature increased from 6 to 50 K. (Blue triangles) Temperature decreased from 50 to 8 K.

Encouraged by the triplet ground state prediction, we irradiated **1** in a frozen toluene solution and employed EPR to characterize the photolysis products. The pulsed Q-band (34 GHz) EPR spectrum recorded at 6 K reveals a characteristic triplet signal,^34,35^ which is attributed to a triplet vinylidene species (Figure 3, left). The spectrum was recorded via free induction decay (FID) to avoid the strong nuclear modulation artifacts found in echo-detected spectra due to the hyperfine (hf) interactions with the ^14^N nuclei in the heterocycle. A continuous wave (CW) X-band (9.6 GHz) EPR spectrum is shown in Figure 3 (right). Spectral simulations assuming an isotropic *g* factor (*g* = 2.0023) yielded the zero-field splitting (ZFS) parameters *D* = +0.377 cm^–1^ and |*E|*/*D* = 0.028.

The positive sign of *D* was directly determined from the thermal polarization of the Q-band EPR spectrum at 6 K (details in Figure S8). Narrow dips observed at 990 mT and 1400 mT in the FID-detected spectrum are due to a reduced pulse EPR intensity at noncanonical field positions, which is typical for triplet species.^36,37^ Temperature-dependent X-band measurements were carried out in the range of 6 to 50 K. A Curie–Weiss plot of EPR intensity vs the reciprocal of temperature^38,39^ (Figure 3, inset) shows a linear dependence, in agreement with the theoretical prediction of a triplet ground state for vinylidene **2**.

The experimentally fitted *D* value of **2** agrees very well with the value computed at the TPSSh/EPR-II level of theory (*D* = +0.373 cm^–1^, Table 1). However, it falls in the range typical for divalent carbenes^41^ (*D* = 0.346–0.409 cm^–1^), so this value alone does not sufficiently constrain the electronic structure of **2**. In contrast, the hyperfine (hf) interaction is expected to be more sensitive to the orbital configuration and should be significantly different for divalent carbenes and vinylidenes (Figure 1). Therefore, we labeled the terminal carbon of **1** with ^13^C (nuclear spin *I* = 1/2) and performed EPR experiments to obtain the ^13^C hf tensor values. In contrast to earlier published results for divalent ^13^C-labeled carbenes,^42−44^ no ^13^C hf splitting was resolved in the field-swept EPR spectrum (Figure S9). This result indicates that the ^13^C hf interaction in **2** is lower than the experimental EPR line width of ∼150 MHz, which stems from the distribution of *D* values (“D-strain”) and unresolved hyperfine interactions with ^14^N.

**Table 1:** ZFS and ^13^C hf Parameters Providing the Best Global Fit to the Q-Band EPR and ENDOR Spectra of **2**^a^

	D	E/D	A_*x*_	A_*y*_	A_*z*_	a_iso_
exp	+0.377	–0.028	57.1	100.0	–7.2	50.0
calc	+0.373	–0.049	51.0	95.0	–12.4	44.5

a The ^13^C hf tensor is assumed to be collinear with the ZFS tensor in the simulation, in agreement with TPSSh/EPR-II DFT calculations. (For more details, see the SI and Figures S12 and S13). All hf values are given in MHz, and *D* is in cm^–1^. The negative sign of *E* was chosen^45^ in accordance with the labeling scheme of the ZFS canonical orientations in Figure 3. The uncertainties in simulated values are ±0.5 MHz for the hf tensor components, ±0.003 cm^–1^ for *D*, and ±0.003 for *E*/*D*.

**Figure 4:**
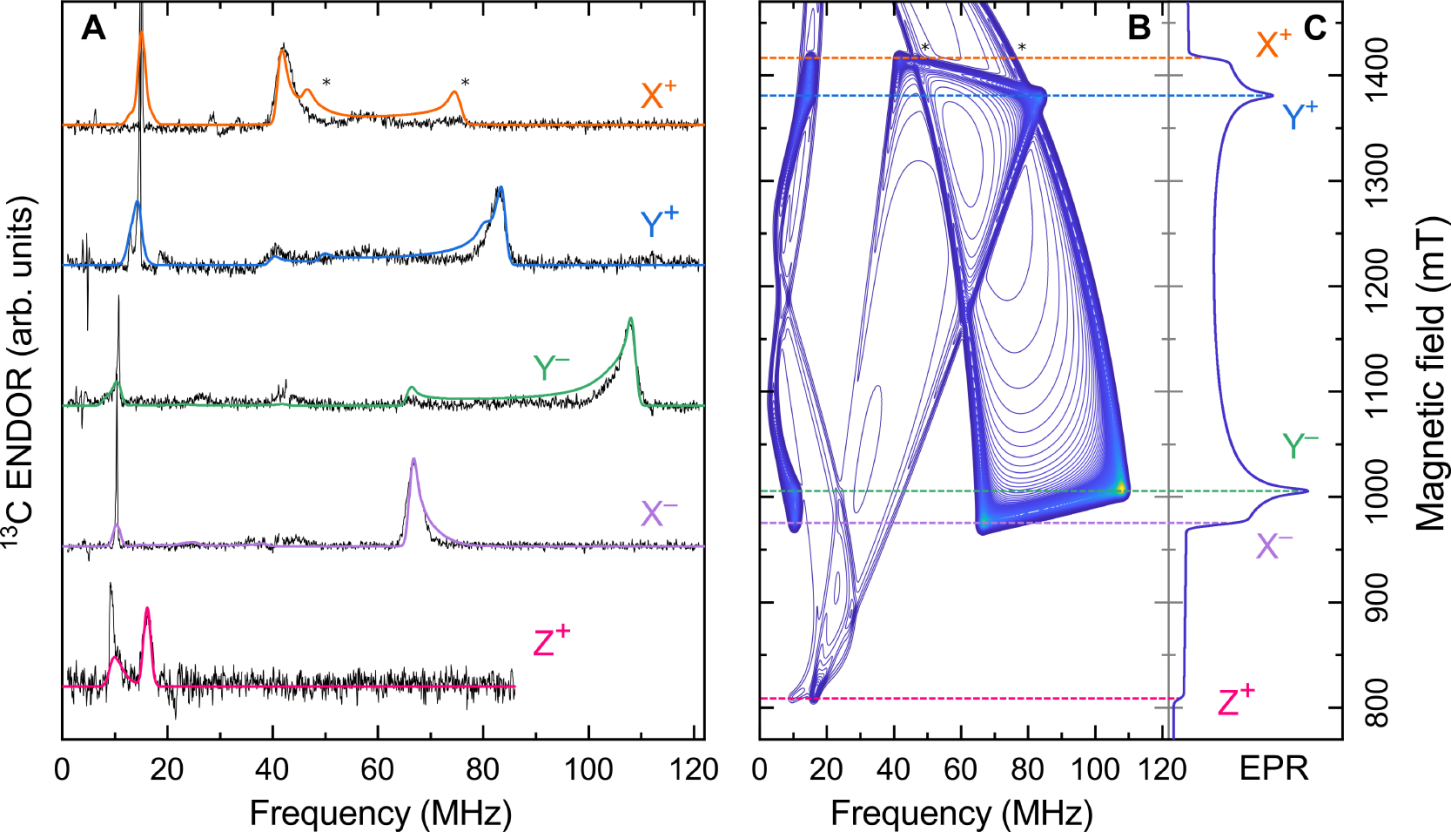
(A) ^13^C Davies ENDOR spectra of **2** acquired at canonical field positions (black) overlaid with simulations (various colors). Asterisks mark simulated ridges originating from the noncanonical orientations. (B) Simulated ^13^C ENDOR pattern derived across the EPR absorption envelope using the global fit parameters (SI). Dashed horizontal lines mark field positions where the ENDOR spectra were recorded. (C) Simulated EPR absorption spectrum.

To resolve the hf tensor, ^13^C-labeled and natural abundance samples of **2** were investigated using the electron–nuclear double-resonance (ENDOR) technique. ENDOR spectra were recorded at five field positions corresponding to the canonical orientations of the ZFS tensor. ^1^H and ^14^N ENDOR signals were suppressed by subtracting the natural-abundance ENDOR spectra (Figure S10), revealing the signals arising solely from ^13^C. Figure 4A shows the orientation-selective ^13^C ENDOR signals of **2** with the corresponding simulations at the canonical magnetic field positions. A good fit was obtained by assuming collinearity of the ZFS and ^13^C hf tensors. Spin Hamiltonian parameters obtained from the global fit are given in Table 1. Simulated ridges marked with asterisks in the X^+^ ENDOR trace originate from the noncanonical ^13^C hf orientations, whose intensity is suppressed in the experiment.^37^

A two-dimensional plot of simulated ^13^C ENDOR signals obtained across the EPR spectrum is depicted in Figure 4B, with the EPR envelope given in Figure 4C. The plot shows the spectral trends for noncanonical ZFS magnetic field positions. We note that the Z^–^ canonical magnetic field position at approximately 1600 mT cannot be reached on a standard Q-band EPR setup equipped with an iron magnet. Therefore, it is not possible to unambiguously determine the ^13^C hf value for the Z orientation. Indeed, the ^13^C ENDOR data at this orientation can be described using either *A*_*z*_ = −7.2 MHz or +24.7 MHz (Figure S11). On the basis of the DFT prediction (see below), *A*_*z*_ = −7.2 MHz was chosen for the simulations shown in Figure 4.

While the ZFS parameters of vinylidene **2** are typical for triplet carbenes, the hf interaction with ^13^C is much weaker than what is usually observed for carbenes (Table 2). The hf tensor can be represented as a sum of its isotropic (*a*_iso_ = [*A*_*x*_ + *A*_*y*_ + *A*_*z*_]/3) and dipolar (*T*_*x*,*y*,*z*_ = *A*_*x*,*y*,*z*_ – *a*_iso_) contributions, where *a*_iso_ is due to the Fermi contact interaction. The decomposition reveals that, in contrast to the dipolar contribution, the *a*_iso_ value of **2** (50 MHz) is significantly lower than those associated with the divalent carbon of triplet carbenes, which range from ∼175 MHz up to ∼260 MHz (Table 2 and Table S1). Similarly, small isotropic ^13^C hyperfine coupling constants have previously been observed in matrix-isolated triplet diradicals such as ^13^CCO (44 MHz)^46,47^ and H^13^CCCH (78.5 MHz),^48^ which however feature much larger *D* values (∼0.6–0.7 cm^–1^)^46,47^ resulting from their distinct electronic structures.

**Table 2:** Comparison of ^13^C hf Tensors for Vinylidene **2** and Representative Triplet Carbenes^a^

triplet species	*T*_*x*_, *T*_*y*_, and *T*_*z*_ (MHz)	*a*_iso_ (MHz)	ref
vinylidene **2**	+7, +50, −57	50^b^	this work
methylene	+30, +24, −54	250	(42)
diphenylcarbene	+17, +41, −58	173	(43)
fluorenylidene	+15, +45, −61	263	(44)

a Note that *A*_*x*,*y*,*z*_ = *a*_iso_ + *T*_*x*,*y*,*z*_, where *a*_iso_ and *T*_*x*,*y*,*z*_ are the isotropic and dipolar parts of the hf tensor.

b For *A*_*z*_ = +24.7 MHz, *a*_iso_ would be 60.6 MHz.

**Figure 5:**
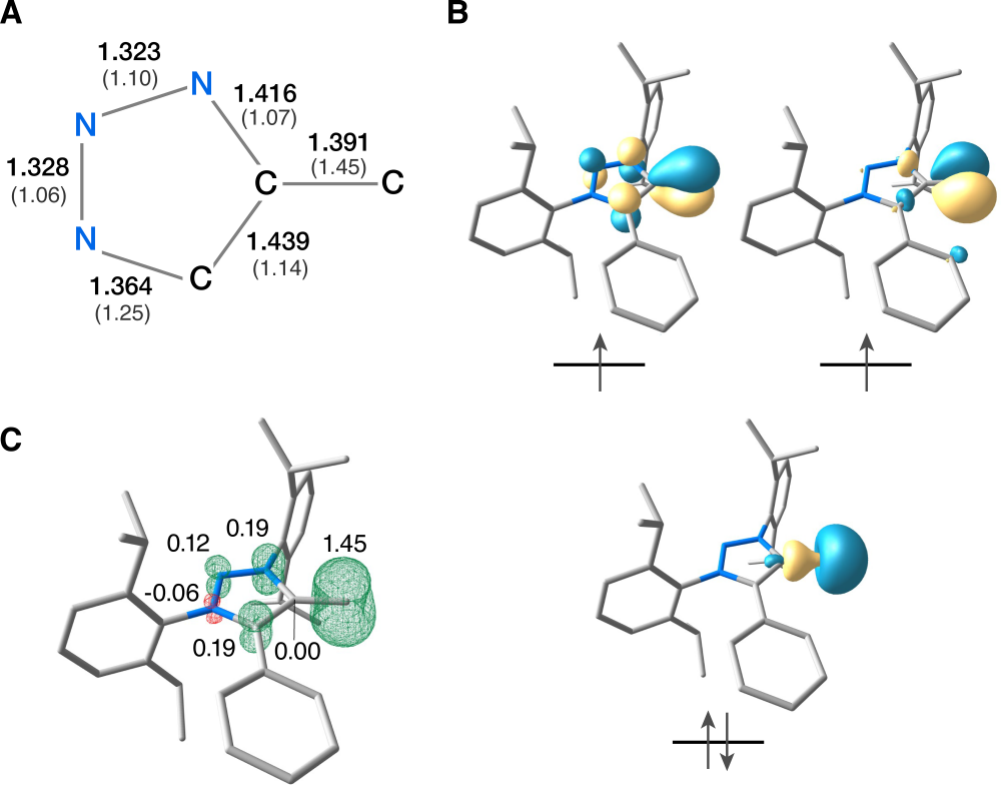
(A) Optimized (B3LYP/def2-TZVP) bond lengths in angstroms and computed Mayer bond orders (in parentheses) of vinylidene **2**. (B) Quasi-restricted orbitals describing the valence electronic structure of the triplet state. (C) Spin density distribution and atomic spin populations for selected atoms, resulting from the TPSSh calculations of EPR parameters.

To rationalize the detected coupling, we investigated the electronic structure of **2** by computational analysis (Figure 5). The terminal C–C bond has intermediate character between a single and double bond (optimized bond length of 1.39 Å, Mayer bond order of 1.45), as a combined result of the strong σ-polarization and π-delocalization. The frontier molecular orbitals indicate that the highest doubly occupied molecular orbital has a significant lone-pair character. One of the two unpaired electrons resides in the nonbonding in-plane p orbital of the monovalent carbon, whereas the other electron occupies an orbital that extends over the π framework and is thus shared between the monovalent carbon and specific members of the heterocycle.^20^ This electronic description is in agreement with a triplet vinylidene in its formal ^3^A_2_ state (Figure 1). Presumably, the π-delocalization, similar to the delocalization in the allyl radical, is related to the stabilization of the triplet state of vinylidene **2**. The resulting spin density has a corresponding π-type profile for the heterocycle and a distinctive toroidal spin distribution around the monovalent carbon, which bears a total spin population of 1.45 electrons.

The TPSSh/EPR-II computed *a*_iso_(^13^C) value of 44.5 MHz for the monovalent carbon agrees with the experimental value (50.0 MHz). The reason for the significantly smaller *a*_iso_ in the triplet vinylidene compared to that in the triplet carbenes is the drastically reduced spin density at the nucleus of the terminal carbon. This is a direct consequence of both singly occupied orbitals of the monovalent carbon having dominant p character, in contrast to the divalent center of triplet carbenes, where the extensive admixture of s character leads to a much stronger Fermi contact term. Unlike *a*_iso_, the dipolar hf components are principally determined by the valence shell as opposed to core spin polarization, which is why, similar to the *D* value, they are less discriminating between triplet carbenes and the present triplet vinylidene (Table 2). Overall, our results demonstrate that *a*_iso_ serves as a definitive spectroscopic signature of triplet vinylidene.

Finally, the stability of triplet vinylidene **2** was assessed by monitoring changes in the EPR intensity at various temperatures. Continuous wave (CW) EPR spectra recorded at X-band (9.5 GHz) directly after irradiation at 82 K and after 1 h revealed no detectable decrease in the EPR intensity (Figure S14A). At 100 K, however, ∼60% of the triplet signal decayed after 1 h (Figure S14B). While the reduction of the EPR intensity at 100 K was nonexponential, the initial 20% of the decay could be simulated with a rate constant of 1.3(1) × 10^–3^ s^–1^ (SI), following the approach described previously.^49^ These data show that triplet vinylidene **2** in toluene is more stable than diphenylcarbene, for which an initial rate constant of 6.7 × 10^–3^ s^–1^ at 99 K has been reported.^49^

## Conclusions

We report the generation and EPR characterization of the first ground-state triplet vinylidene. While the zero-field splitting parameters are similar to those of triplet carbenes, the isotropic ^13^C hyperfine coupling is far outside of the classical range, demonstrating a unique class of molecules with a distinct electronic structure. The discovery of a new class of high-spin-state compounds, featuring a monovalent carbon atom, opens up a completely new field of chemistry. To date, only fleeting singlet vinylidenes have been considered, while triplet vinylidenes remain overlooked. However, by inducing bond polarization through an ylidic/mesoionic system and the delocalization of spin density, the triplet state can become the ground state. Obviously, the reactivity and stability of this new molecule class are completely unexplored. Triplet carbenes and nitrenes have been thoroughly investigated with a long list of examples; therefore, the search for new, more stable, maybe even room-temperature-stable triplet vinylidenes has now started. Since high-spin ground-state species not only possess interesting electronic structures but also have found applications in magnetic materials or spintronics,^50,51^ the exploration of this overlooked compound class might reveal opportunities for exciting applications.

## Supporting Information Available

Experimental procedures, characterization data, spectra, EPR data, and computational details (PDF)
