# Supplementary material for: Characterization of a Triplet Vinylidene
Source: J Am Chem Soc. 2021 Dec 13;143(50):21410–5. doi: 10.1021/jacs.1c11062 (PMC8704171; doi:10.1021/jacs.1c11062)
Supplement: Supplementary file 1 — ja1c11062_si_001.pdf [file ja1c11062_si_001.pdf]

Supplementary Materials for

Characterization of a Triplet Vinylidene

Yury Kutin,[a] Justus Reitz,[a] Patrick W. Antoni,[a] Anton Savitsky,[b] Dimitrios A. Pantazis,*[, c] Müge Kasanmascheff,*[, a] Max M. Hansmann*[, a]

a) Department of Chemistry and Chemical Biology, Technische Universität Dortmund, Otto-Hahn-Str. 6, 44227 Dortmund, Germany;
b) Department of Physics, Technische Universität Dortmund, Otto-Hahn-Str. 4a, 44227 Dortmund, Germany.
c) Max-Planck-Institut für Kohlenforschung, Kaiser-Wilhelm-Platz 1, 45470 Mülheim an der Ruhr, Germany.

Correspondence to: dimitrios.pantazis@kofo.mpg.de; muege.kasanmascheff@tu-dortmund.de; max.hansmann@tu-dortmund.de

Table of Contents

1. Materials and Methods (Synthesis)... 2
2. Characterization data/NMR irradiation experiment.. 2
3. Quantum chemical calculations 7
   3.1. Computational details 7
   3.2. Computed singlet-triplet gaps 9
4. EPR data.. 10
   4.1. EPR sample preparation.. 10
   4.2. EPR experimental details... 10
   4.3. Determination of the sign of *D* 11
   4.4. EPR line shape comparison for a ^{13}C-labeled and natural abundance sample 12
   4.5. Subtraction of ^{1}H and ^{14}N ENDOR signals 13
   4.6. Sign of the A_z component of the ^{13}C hf tensor 14
   4.7. ENDOR simulations based on the spin Hamiltonian parameters derived from DFT and the best fit to the data 15
   4.8. Comparison of the vinylidene and carbene spin Hamiltonian parameters 18
   4.9. Stability of the triplet vinylidene 19
5. References... 20

1. Materials and Methods (Synthesis)

All solvents were purified with a MBraun SPS – 800 and additionally dried over molecular sieves and degassed with argon. Reactions were carried out either under N_2 or Ar atmosphere. Solids were handled and NMR and EPR samples were prepared in a nitrogen-filled glovebox. High resolution MS (EI): Finnigan MAT 8200 (70 eV), ESI-MS: Finnigan MAT 95, accurate mass determinations: Bruker APEX III FT-MS (7 T magnet) and LTQ-Orbitrap-XL (Thermo Scientific) equipped with a heated electrospray ionization source (HESI). NMR: NMR spectra were measured on the spectrometers Bruker AV 500 Avance NEO, Bruker AV 400 Avance III HD NanoBay, AV 600 Avance III HD and AV 700 Avance III HD, and chemical shifts (δ) are referenced to their solvent signals [C_6D_6, 7.16 (^{1}H NMR) 128.06 (^{13}C NMR)], coupling constants (*J*) are in Hz. All spectra were recorded in 5 mm NMR tubes at the temperatures indicated. The solvent signals were used as references and the chemical shifts converted to the TMS scale. UV-Vis spectra were recorded on an Agilent Cary60. All commercially available compounds (Acros, ABCR, Alfa Aesar, Sigma Aldrich, Fluorochem) were used as received. IR-ATR measurements (diamond) were performed in reflection mode on a Bruker Alpha II inside a glovebox, wavenumbers are in cm^{-1}. Melting points were measured with a Büchi M-560 apparatus.

Diazoalkene **1a** and ^{13}C-labelled **1a** were prepared as described in the literature.[1]

2. Characterization data/NMR irradiation experiment

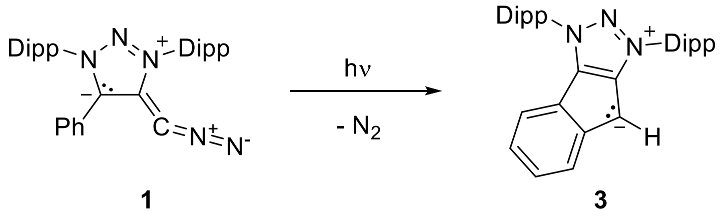

J-Young NMR experiment: A ^{1}H NMR spectrum of a C_6D_6 (0.5 mL) solution of **1** (20 mg) was obtained. The solution was irradiated with a Kessil LED (390 nm) at a distance of ca. 20 cm for 30 min. Another ^{1}H NMR spectrum was directly measured indicating the formation of **3**.

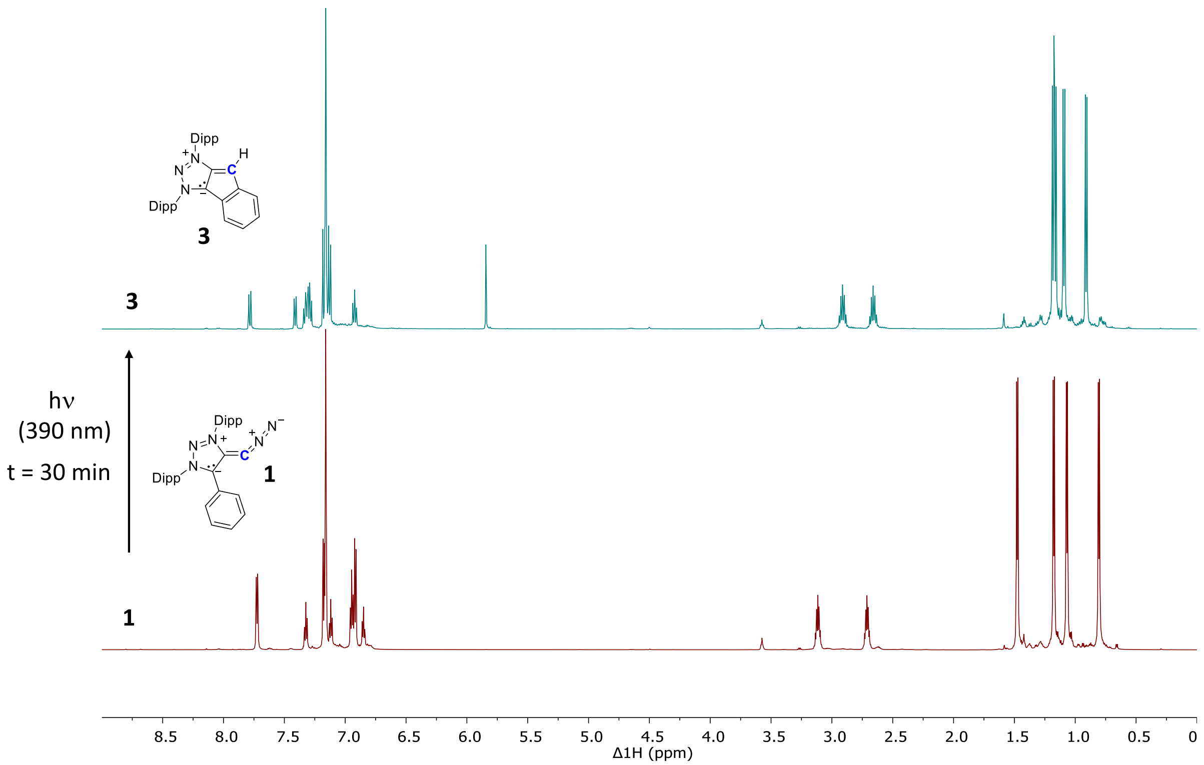

Figure S1. ^{1}H NMR spectrum (C_6D_6, 700 MHz, 298 K) of **1** (bottom) followed by irradiation at 390 nm and remeasurement (top, 500 MHz) to give CH-insertion product **3**.

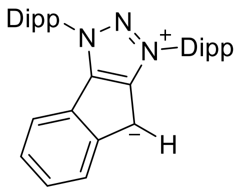

Under an atmosphere of argon, a solution of **1** (101 mg, 0.2 mmol, 1.0 eq.) in toluene (15 mL) at ambient temperature was irradiated with a 390 nm Kessil lamp (10 cm distance) for 1 h. A color change from orange to red-pink was observed. The solvent was removed under reduced pressure. The resulting solid was extracted with pentane (2 x 8 mL) and the solvent removed under reduced pressure. Filtration over celite and crystallization from pentane at -40 °C furnished **3** (71 mg, 0.15 mmol, 75%) as a red-pink solid.

m.p. 132 °C decomp.; **^{1}H NMR** (500 MHz, C_6D_6): δ [ppm] = 7.78 (d, *J* = 8.3, 1H, Ar-H), 7.43 – 7.39 (m, 1H, Ar-H), 7.37 – 7.25 (m, 3H, Ar-H), 7.17 (d, *J* = 8.0 Hz, 2H, Ar-H), 7.12 (d, *J* = 7.8 Hz, 2H, Ar-H), 6.93 (ddd, *J* = 8.0, 6.9, 1.0 Hz, 1H, Ar-H), 5.84 (s, 1H, C-H), 2.92 (hept, *J* = 6.9

Hz, 2H, C*H*(CH_3)$_2$), 2.66 (hept, *J* = 6.9 Hz, 2H, C*H*(CH_3)$_2$), 1.18 (d, *J* = 6.9 Hz, 6H, C*H*(CH_3)$_2$), 1.17 (d, *J* = 6.9 Hz, 6H, C*H*(CH_3)$_2$), 1.09 (d, *J* = 6.9 Hz, 6H, C*H*(CH_3)$_2$), 0.91 (d, *J* = 6.8 Hz, 6H, C*H*(CH_3)$_2$); **^{13}C NMR** (126 MHz, C_6D_6): δ [ppm] = 147.5 (Ar-C_q), 147.4 (Ar-C_q), 146.5 (Ar-C_q), 141.4 (Ar-C_q), 133.3 (Ar-C_q), 133.0 (Ar-C_q), 131.6 (Ar-C_q), 131.2 (Ar-CH), 129.5 (Ar-C_q), 124.7 (Ar-CH), 124.4 (Ar-CH), 124.4 (Ar-CH), 120.9 (Ar-CH), 119.1 (Ar-CH), 115.1 (Ar-CH), 108.3 (Ar-C_q), 68.8 (CH), 29.1 (*C*H(CH_3)$_2$), 29.1 (*C*H(CH_3)$_2$), 24.8 (CH(*C*H_3)$_2$), 24.6 (CH(*C*H_3)$_2$), 23.9 (CH(*C*H_3)$_2$), 23.5 (CH(*C*H_3)$_2$); **IR** [cm^{-1}]: ṽ =2867, 1525, 1458, 1363, 1318, 1274, 1235, 1214, 1158, 1100, 1061, 983, 807, 758, 740, 648, 429; **HR-MS-ESI(+)** calc. $C_{33}H_{40}N_3^+$ 478.3222 $[M+H]^+$; 478.3219 found.

NMR spectra

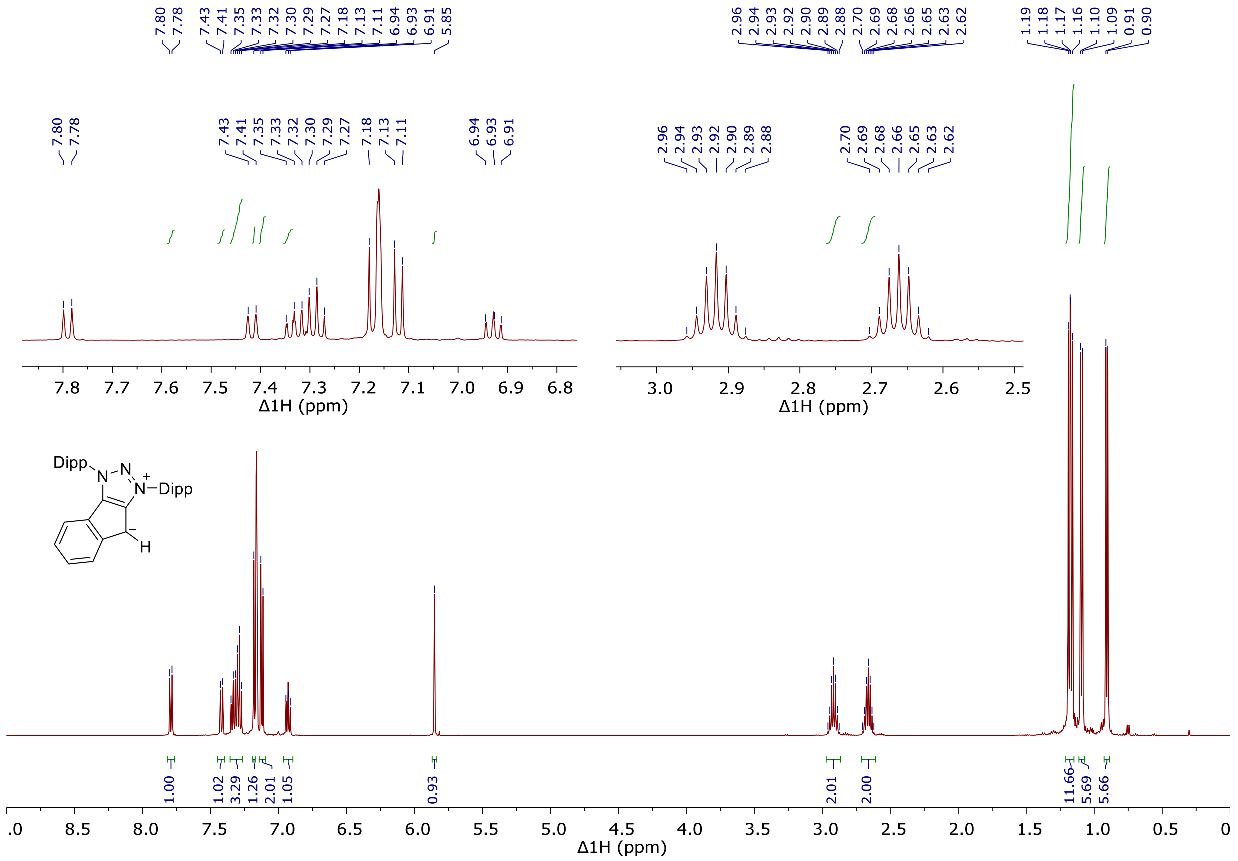

Figure S2. ^{1}H NMR (500 MHz, C_6D_6, 298K) of **3.**

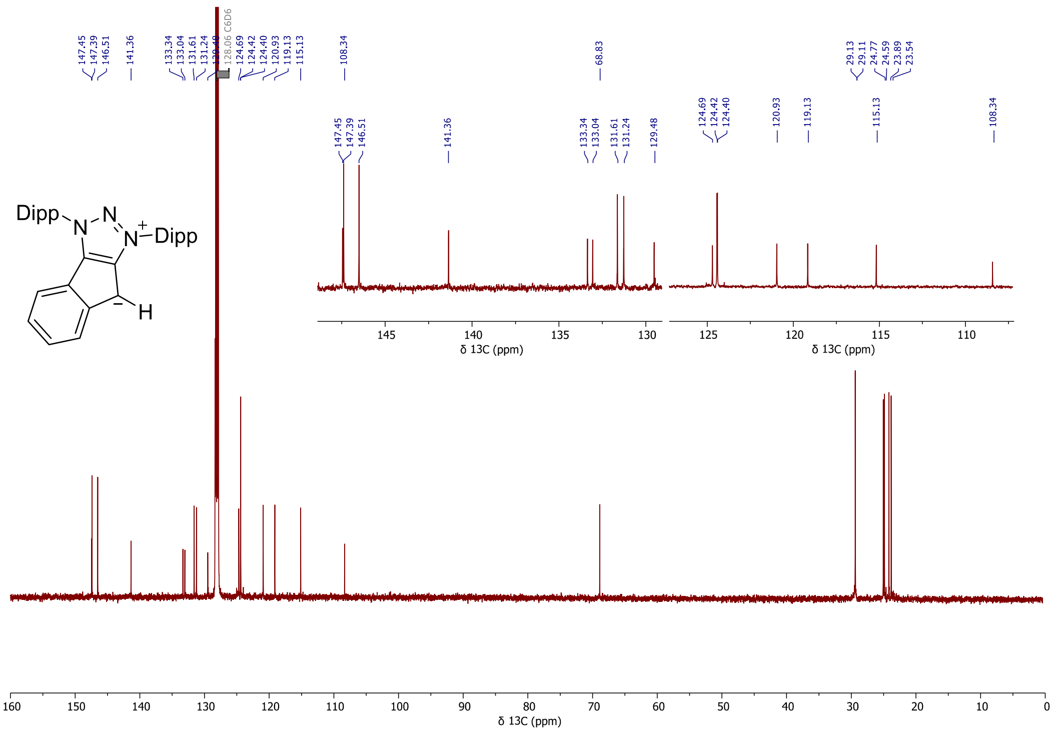

Figure S3. ^{13}**C {**^{1}**H} NMR** (126 MHz, C_6D_6, 298K) of **3**.

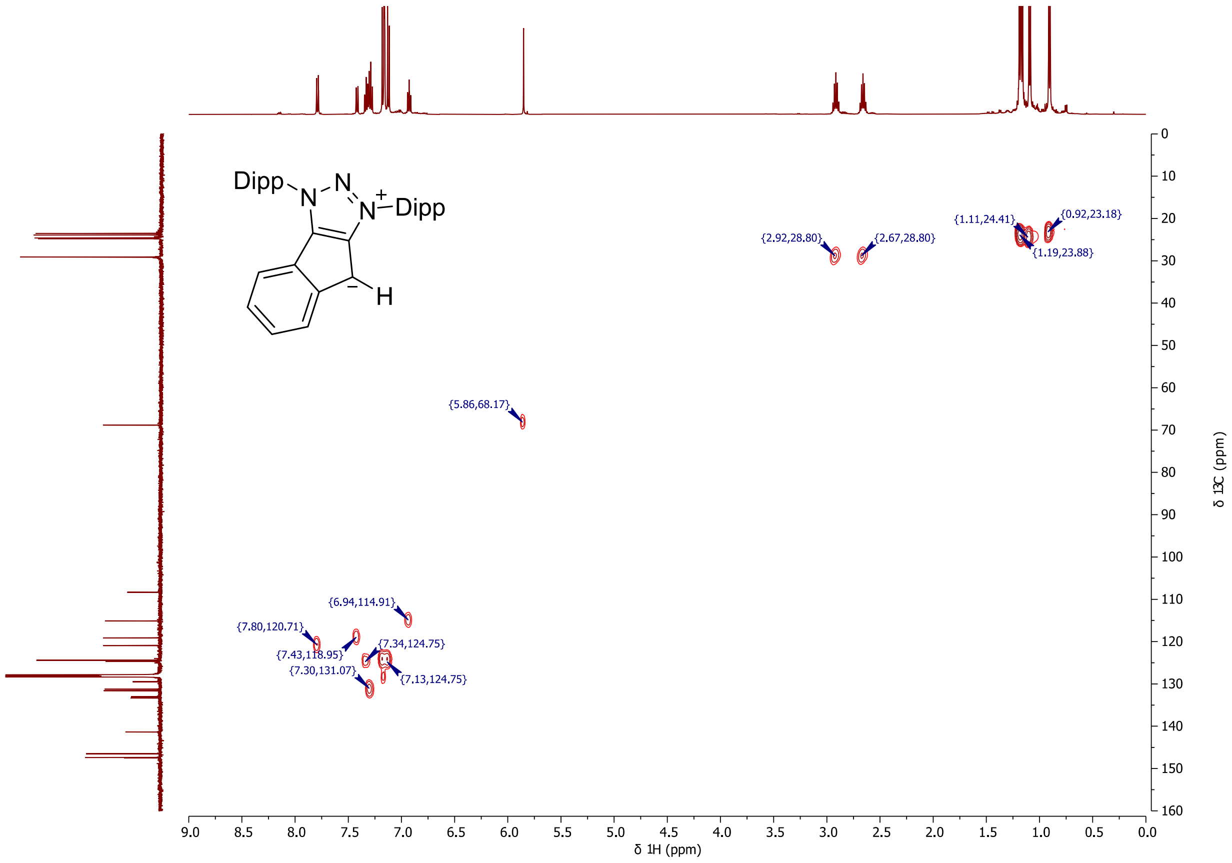

Figure S4. ^{1}**H/**^{13}**C HSQC** (500/126 MHz, C_6D_6, 298K) of **3**.

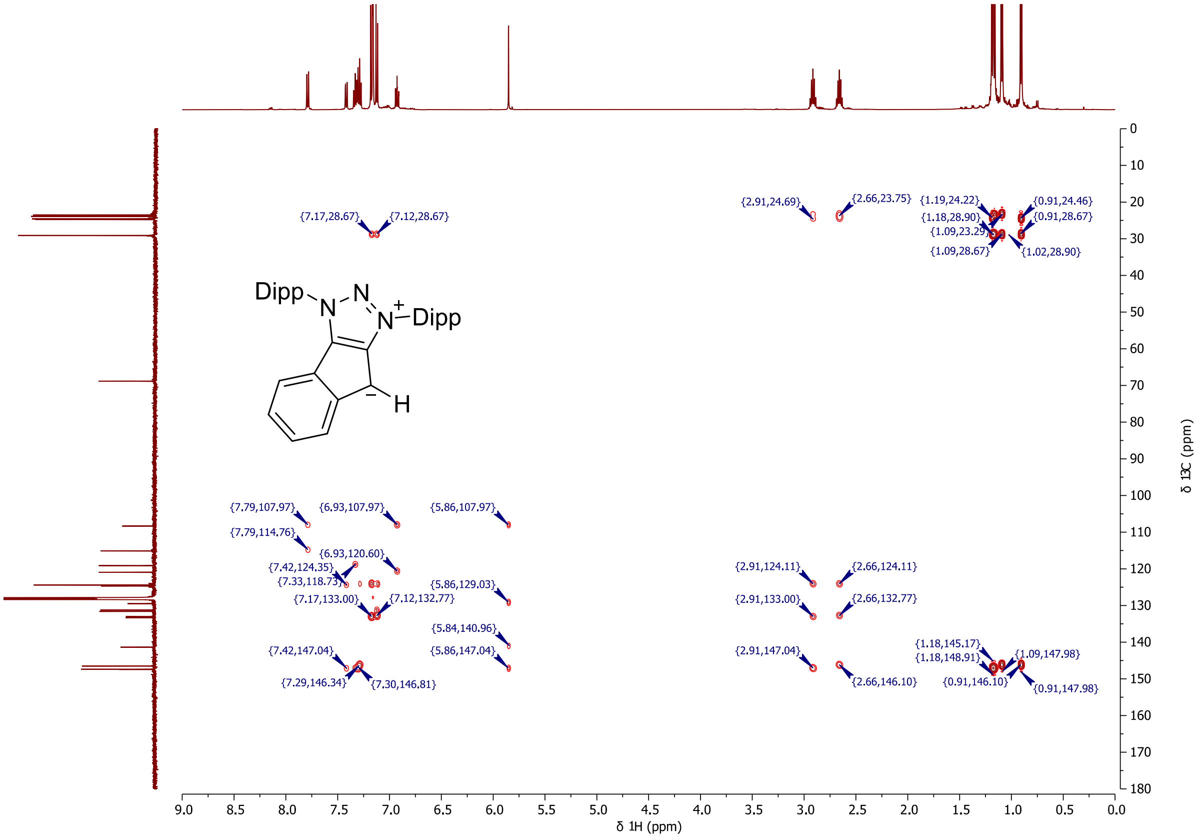

Figure S5. ^{1}**H/**^{13}**C HMBC** (500/126 MHz, C_6D_6, 298K) of **3**.

3. Quantum chemical calculations

3.1. Computational details

Geometries

All quantum chemical calculations in the present work were performed with the Orca package.[2] Geometries of vinylidene **2** and of a simplified model where all ring substituents were replaced by hydrogen atoms were optimized in both spin triplet and singlet states with the B3LYP functional.[3-4] The def2-TZVP basis sets were used on all atoms.[5] A fully decontracted version of the auxiliary def2/J basis set[6] was employed for fitting of the Coulomb integrals, while the chain-of-spheres approximation[7] was used for exact exchange. Tight convergence criteria were used both for the energy and the gradient, while significantly increased integration grids were applied throughout (Grid6 and GridX8 in Orca convention) to ensure elimination of numerical errors. Using state-specific optimized geometries of the singlet and triplet states of vinylidene **2**, the triplet minimum is predicted to be 7.2 kcal/mol lower than the singlet minimum at the B3LYP/def2-TZVP level.

EPR parameters

The triplet ground state geometry was used for single-point calculations of the zero-field splitting (ZFS) parameters, i.e. the D tensor, and of ^{13}C hyperfine coupling constants (HFCs), i.e. the A tensors. EPR calculations were performed with the TPSSh functional,[8] which is known from extensive experience to be one of the best choices for EPR parameters, particularly for hyperfine coupling tensors of challenging systems.[9-13] Comparison with B3PW91, an alternative choice that performs well for HFCs in some compound classes,[14] showed negligible differences. A more important parameter concerns the choice of basis set for the calculation of hyperfine coupling constants (HFC), because of the increased requirements in the description of spin polarization and the spin density distribution close to the nucleus. For this reason the purpose-made EPR-II basis set[15] was used on selected atoms for HFC calculations, while other atoms were still described with the def2-TZVP basis set. An alternative choice of basis set optimized for the calculation of HFCs is the pcH-2 basis set by Jakobsen and Jensen.[16] We confirmed that the two basis sets agree on ^{13}C A_{iso} values within 0.5 MHz. The A tensor calculations included the isotropic (Fermi contact) and dipolar contribution for the ^{13}C nucleus. D tensor calculations included both spin–spin coupling (D_{SSC}) and spin–orbit coupling (D_{SOC}) contributions to the ZFS. The spin–spin contributions were computed on the basis of unrestricted natural orbitals (UNOs).[17-18] The spin–orbit components were derived with the coupled-perturbed approach[19] using a mean-field approximation to the Breit–Pauli operator as an effective potential spin–orbit coupling operator.[20-22]

Correlated wave function based calculations

Going beyond DFT, we used the domain-based local pair natural orbital implementation of coupled cluster theory with singles, doubles, and perturbative triples excitations, DLPNO-

CCSD(T).[23-25] This method provides results that reproduce closely the results of canonical CCSD(T), which is considered the "gold standard" of modern quantum chemistry. The DLPNO-CCSD(T) approach was also used recently as the benchmark method to produce reference-quality spin-state energetics and electronic structure analysis of aryl-carbenes.[26-28] For the present purposes we used the "TightPNO" collective setting in Orca, the highest available default setting. In addition, we utilized the "T1" approximation for the perturbative triples component as opposed to the less reliable "T0" approach.[29] For both spin states Kohn–Sham orbitals were used as initial input for the DLPNO procedure.

Both DFT and DLPNO-CCSD(T) are single-reference approaches that compare the ground state triplet with a closed-shell excited singlet state, but by definition they cannot address the possibility of an "open-shell" singlet state. Therefore, as a final level of refinement we performed complete active space self-consistent field (CASSCF) calculations utilizing a complete active space of 10 electrons distributed within 8 valence orbitals, i.e. CAS(10,8). The capability of Orca to average over states of different spin multiplicity was leveraged to perform state-averaged orbital optimization over the lowest triplet and the two lowest singlet states simultaneously. The resulting orbitals (see Figure S6) were subsequently used in a multireference perturbation theory treatment using the N-electron valence state perturbation theory, NEVPT2,[30-31] to include dynamic electron correlation. All wave function based methods were applied on a simplified model of vinylidene **2** where the ring substituents were replaced with hydrogens. The def2-TZVP basis sets were used throughout.

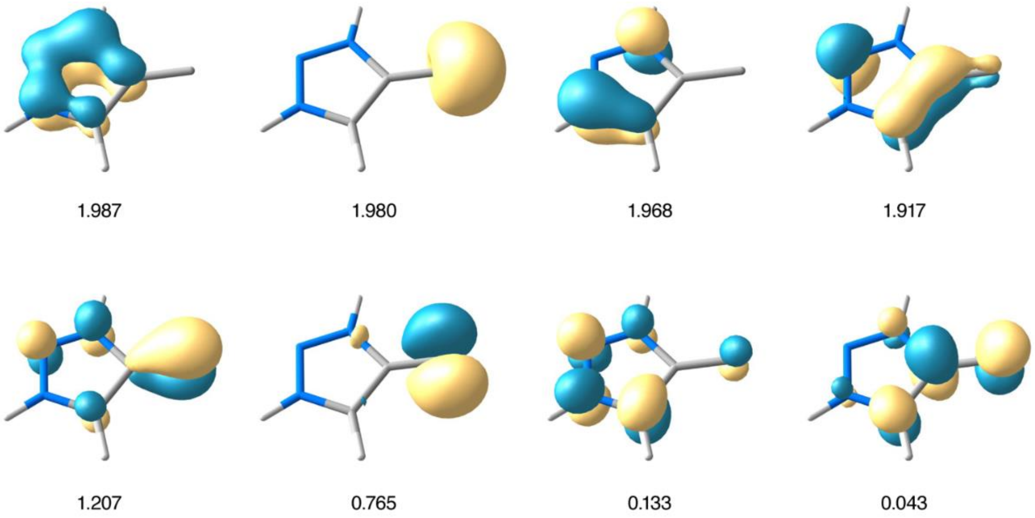

Figure S6. Orbitals and natural occupation numbers produced from CASSCF(10,8) calculations on the simplified model of vinylidene **2**. The orbital optimization and resulting occupation numbers are obtained by state-averaging over the triplet ground state as well as the two lowest excited singlet states (closed-shell and open-shell singlet) simultaneously.

3.2. Computed singlet-triplet gaps

The inherent spin-state energetics of the simplified (H-substituted) heterocyclic system of vinylidene **2** were probed at three different levels of theory. First, DFT calculations with the B3LYP functional yield a preference of 14.3 kcal/mol for the triplet state (adiabatic energy difference: 12.2 kcal/mol), compared to the lowest singlet state in which the non-bonding in-plane orbital of the monovalent carbon is unoccupied (see Figure 5). To address potential biases of DFT, at a second level of refinement we used the most accurate implementation of the domain-based local pair natural orbital implementation of coupled cluster theory with singles, doubles, and perturbative triples excitations, DLPNO-CCSD(T1).[23-25, 29] This provides a vertical energy gap of 13.1 kcal/mol for the model system in favor of the triplet state.

Finally, we turned to multireference calculations, which allow us to also probe the "open-shell" singlet state. CASSCF and NEVPT2 place the closed-shell singlet at 12.0 kcal/mol and 12.2 kcal/mol, respectively, above the ground state triplet state. The open-shell singlet state, with the same orbital occupation as the triplet ground state, is predicted to be at 17.0 kcal/mol by CASSCF and at 13.6 kcal/mol by NEVPT2.

In conclusion, all levels of quantum chemical treatment concur that the excited singlet states, regardless of their exact electronic character, are more than 12 kcal/mol above the ground state triplet state for the simplified model of the vinylidene (Figure S7). The exceptional agreement of the DFT values with both DLPNO-CCSD(T) and NEVPT2 results give strong confidence in the reliability of the singlet-triplet gap predicted by DFT also for the full model of vinylidene **2**.

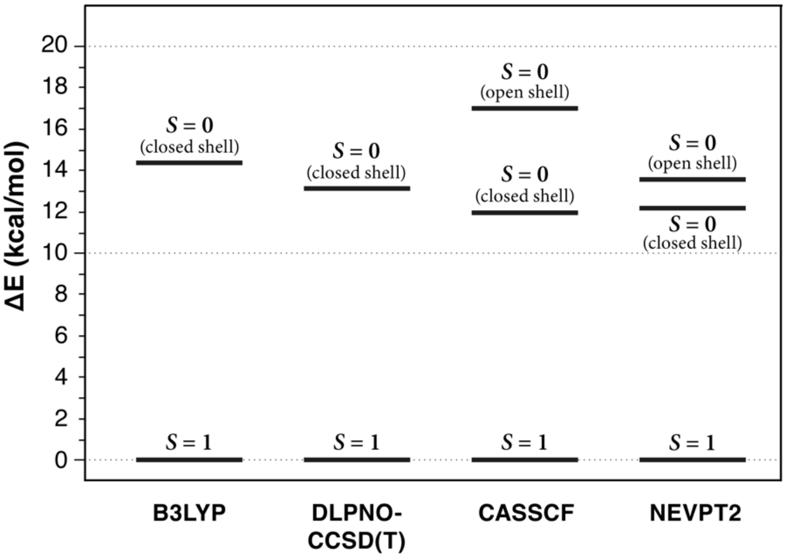

Figure S7. Vertical energy differences between the ground triplet state and the excited singlet states obtained from different levels of theory on the simplified model of vinylidene **2**. All calculations were performed with the def2-TZVP basis set.

4. EPR data

4.1. EPR sample preparation

All samples were prepared in a nitrogen-filled glovebox. For Q-band measurements a 20 ± 1 mM solution of **1** in thoroughly degassed, dry toluene was transferred into a 1.6 mm quartz tube (1.5 cm filling height), cooled to -40 °C for 15 min and sealed with Critoseal (3-5 mm). For X-band measurements a 6 ± 1 mM solution of **1** in thoroughly degassed, dry toluene was transferred into a J-Young capped 4 mm quartz tube (1.5 cm filling height) and closed at room temperature. The samples were covered in aluminum foil inside the glovebox and transported to the spectrometer under exclusion of light.

4.2. EPR experimental details

Q-Band pulse EPR measurements were carried out at 6 K using a Bruker Elexsys E580 spectrometer equipped with a 150 W TWT amplifier, Bruker EN 5107D2 resonator, Oxford Instruments CF935 continuous-flow helium cryostat and Oxford Instruments MercuryiTC temperature controller. Field-swept EPR spectra were detected via the free induction decay (FID) signal to avoid strong nuclear modulation artifacts found in the electron spin echo detected spectra. The microwave (MW) π/2 pulse was 500 ns. The triplet species was generated by irradiating the diazo precursor (20 mM) in frozen toluene solution at 10 K using a Hg arc lamp (LOT LSB610U) inside the resonator for 1 hour.

Orientation-selective Davies ENDOR spectra were collected at 6 K using an AR 600 W radiofrequency (RF) amplifier (AR 600A225A). The following microwave pulse sequence was used: π−T−π/2−τ−π−τ−echo. The RF pulse was applied during the time interval T and had a length of 30 μs; the MW inversion π pulse was 28−30 ns; the π/2 and π detection pulses were 14 and 28 ns, respectively; the inter-pulse delay τ was 340 ns.

Temperature-dependent X-band continuous wave (CW) EPR measurements were carried out in the range of 6 to 50 K using a Bruker Elexsys E500 spectrometer equipped with a Bruker ER 4116DM resonator, Oxford Instruments ESR 900 cryostat and Oxford Instruments MercuryiTC temperature controller. The spectra were recorded under non-saturating conditions, with a modulation amplitude of 7 G. The triplet species was generated by irradiating the diazo precursor (6 mM) in frozen toluene solution at 7 K using a Xe arc lamp (LOT-QuantumDesign, 300W) inside the resonator for 1 hour.

X-Band CW EPR measurements for the stability study were carried out using a Bruker Elexsys E500 spectrometer equipped with a Bruker ER 4122SHQE resonator and ER 4141VT variable temperature accessory. The spectra were recorded under non-saturating conditions (0.2 mW) in the temperature range 82 – 100 K; the modulation amplitude was 15 G. The triplet species was

generated by irradiating the diazo precursor (6 mM) in frozen toluene solution at 82 K using a UV diode (λ = 395 nm) inside the resonator for 30 minutes.

EPR and ENDOR simulations were performed using the EasySpin package[32]. For the 2D ^{13}C ENDOR simulations the magnetic field was varied from 770 to 1650 mT in increments of 0.3 mT, and the frequency was varied from 0 to 125 MHz in increments of 125 kHz. The *salt* function was used with an excitation width parameter *Exp.ExciteWidth* = 84 MHz, EPR broadening *Sys.HStrain* = [160 120 160] MHz, ENDOR linewidth *Sys.lwEndor* = 1.6 MHz and *Opt.GridSize* = [185 3].

4.3. Determination of the sign of *D*

The sign of D can be determined from the intensity difference between the $M_S = -1 \leftrightarrow 0$ and $M_S = 0 \leftrightarrow 1$ transitions at low temperatures. The former has a higher intensity than the latter due to the Boltzmann distribution of the electronic M_S states populations (see Figure S8). In the triplet EPR spectrum of **2** recorded at 6 K, the canonical field positions X^- and Y^- are found on the low-field side, indicating $D > 0$ (see Figure 3 and Figure S9).

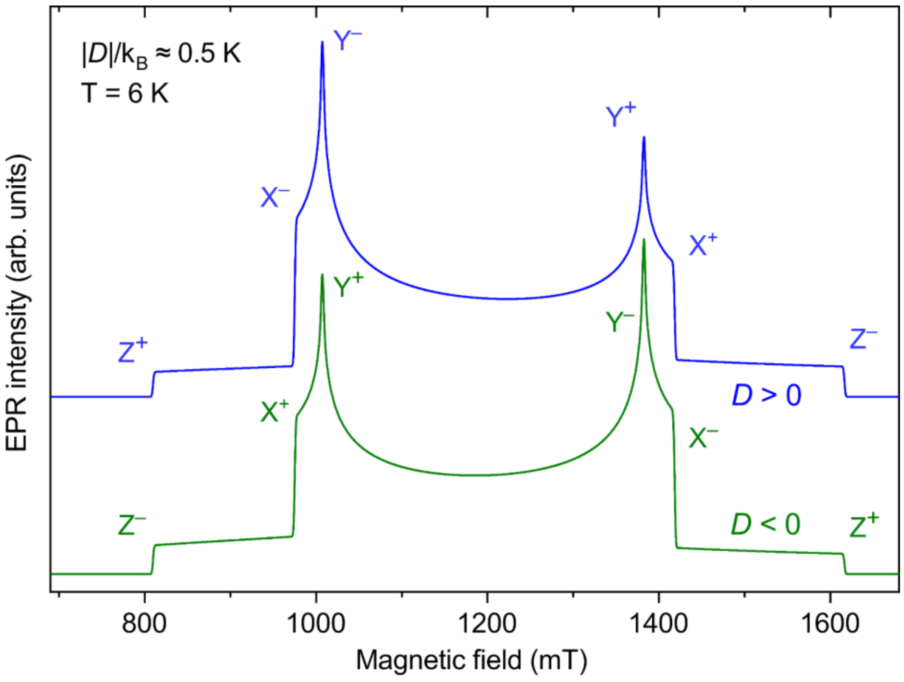

Figure S8. Simulated EPR spectra for a positive and negative ZFS D value. $|D|$ = 0.377 cm^{-1} and $|E/D|$ = 0.028; T = 6 K, f_{MW} = 34 GHz.

4.4. EPR line shape comparison for a ^{13}C-labeled and natural abundance sample

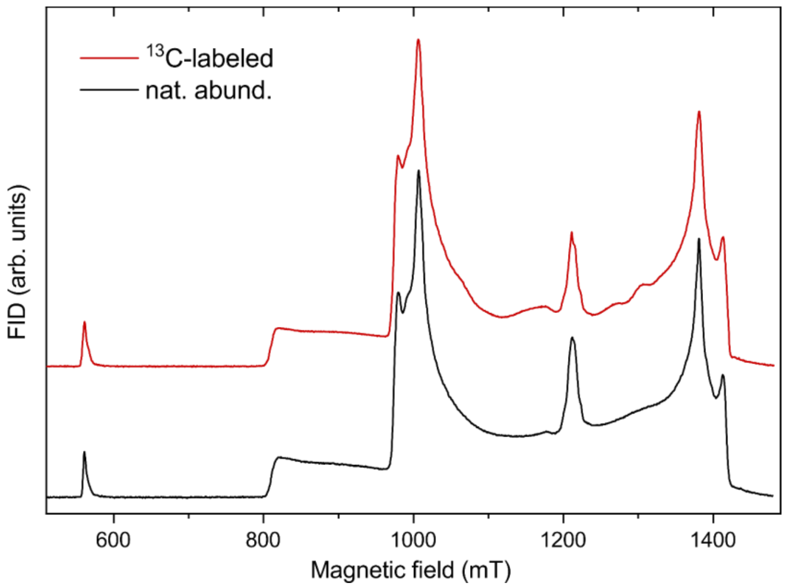

Figure S9. FID-detected Q-band ($f_{MW} \approx 33.9$ GHz) EPR spectra of **2** in toluene collected at 6 K for a ^{13}C-labeled (red) and natural abundance (black) sample. No hf splitting is observed in the field-swept EPR spectrum upon ^{13}C labeling.

4.5. Subtraction of ^{1}H and ^{14}N ENDOR signals

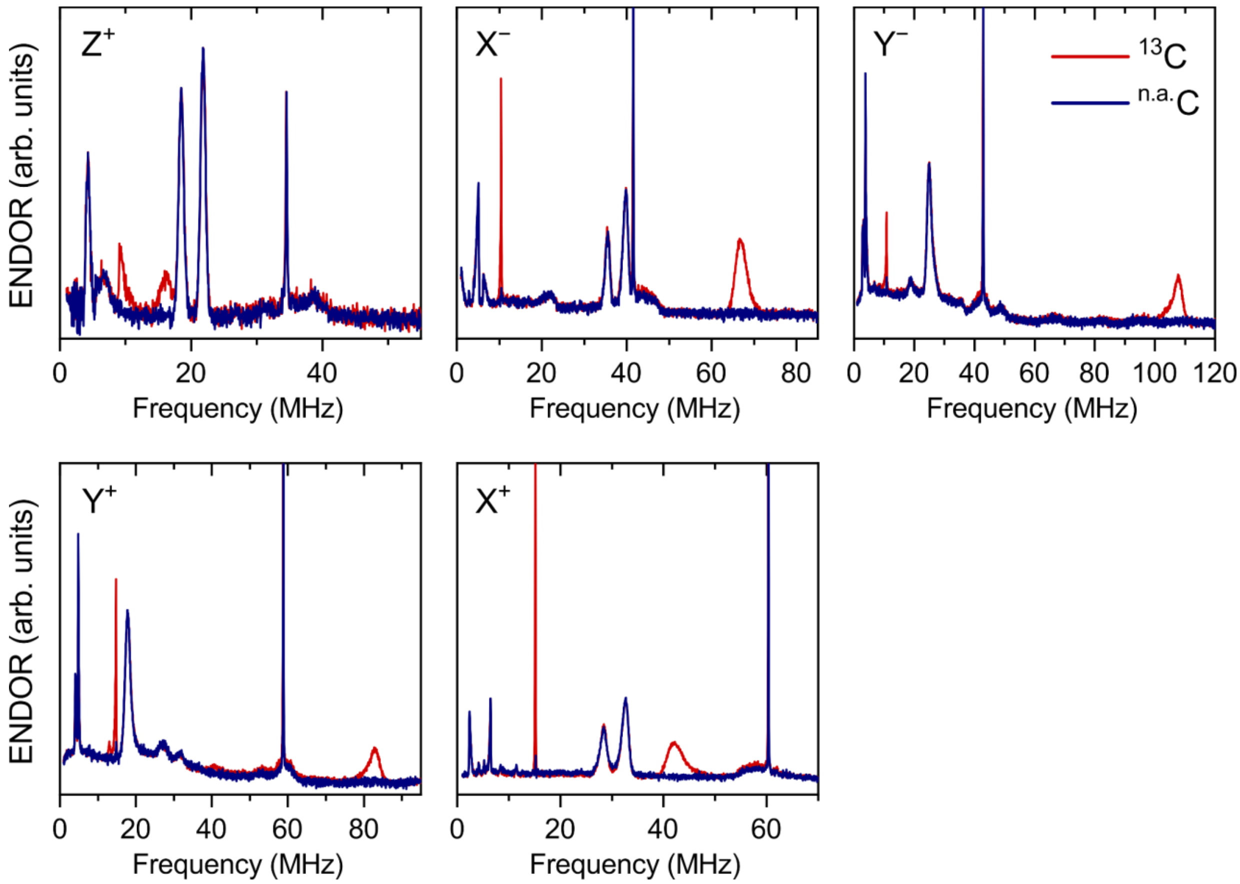

Figure S10. Davies ENDOR spectra of **2** recorded at canonical field positions for a ^{13}C-labeled (red) and a natural abundance (dark blue) sample. Subtraction results for each field position are shown in Figure 4 and Figure S11.

4.6. Sign of the A_z component of the ^{13}C hf tensor

For a standard Q-band setup with a resistive magnet, magnetic field is limited by 1450 mT. Thus, the Z^- canonical field position (~1600 mT) cannot be probed experimentally. This creates an uncertainty in the experimentally determined A_z component of the ^{13}C hf tensor, as two values (a negative −7.2 and positive +24.7 MHz) can describe the available experimental data. $A^{exp}_z = -7.2$ MHz was chosen based on a close match with the DFT-derived value of $A^{DFT}_z = -11.4$ MHz.

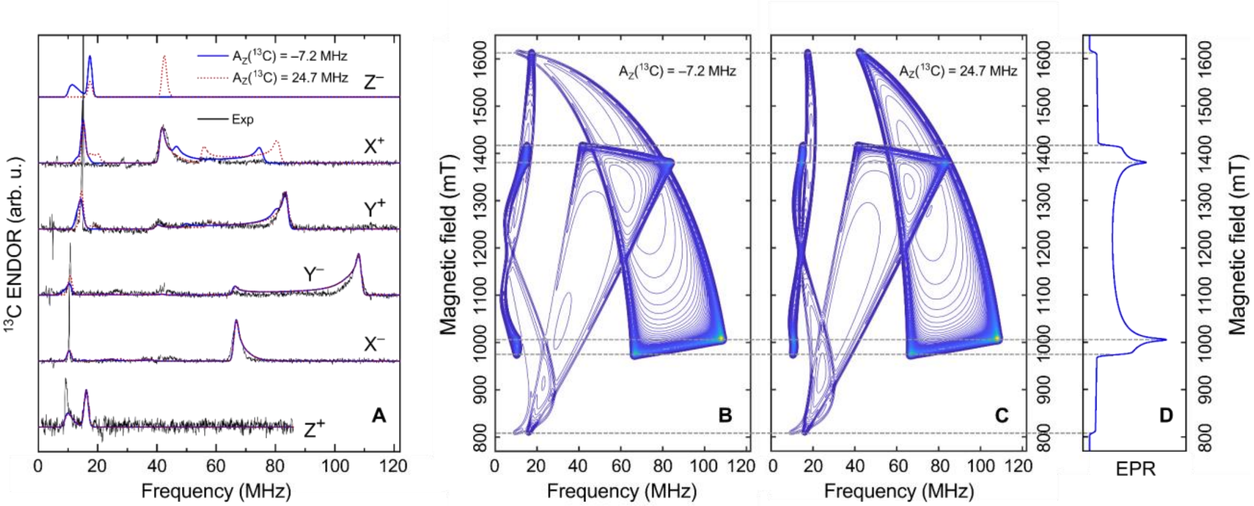

Figure S11. (**A**) ^{13}C Davies ENDOR signals of **2** recorded at 6 K at canonical field positions (black lines) overlaid with simulations obtained for a negative (blue solid lines) and positive (red dotted lines) value of the A_z component of the ^{13}C hf tensor. As the magnetic field is limited by 1450 mT, only simulated traces are given for the Z^- field position. The experimental traces represent difference between the spectra of a ^{13}C-labeled and natural abundance sample. (**B** and **C**) Simulated ^{13}C ENDOR patterns derived across the full EPR absorption envelope using the global fit parameters with $A_z = -7.2$ and $A_z = +24.7$ MHz, respectively (other spin-Hamiltonian parameters were identical, see Table 1). Dashed horizontal lines mark the canonical field positions. (**D**) Simulated EPR spectrum of **2**.

4.7. ENDOR simulations based on the spin Hamiltonian parameters derived from DFT and the best fit to the data

The ZFS tensor (in cm^{-1}) calculated by Orca in the molecular frame, where the *z* axis is along the C–C bond, *x* is perpendicular to the plane of the heterocycle and *y* is in the plane of the heterocycle, perpendicular to both *x* and *z* (see Figure S12) is given as

$$\widehat{D} = \begin{pmatrix} 0.028430 & 0.000706 & 0.002613 \\ 0.000706 & 0.065032 & -0.008072 \\ 0.002613 & -0.008072 & 0.419323 \end{pmatrix}.$$

Diagonalization of the $\widehat{D}$ matrix yields the eigenvalues and a matrix of the corresponding right column eigenvectors:

$$\widehat{D}_{diag} = \begin{pmatrix} 0.0284 & 0 & 0 \\ 0 & 0.0649 & 0 \\ 0 & 0 & 0.4195 \end{pmatrix},\ R_D = \begin{pmatrix} 0.9998 & 0.0210 & 0.0066 \\ -0.0208 & 0.9995 & -0.0228 \\ -0.0071 & 0.0226 & 0.9997 \end{pmatrix}.$$

A subsequent subtraction of $(D_{xx}+D_{yy}+D_{zz})/3 = 0.1842\ cm^{-1}$ from each of the ZFS eigenvalues yields the traceless tensor:

$$\widehat{D}_{traceless} = \begin{pmatrix} -0.1425 & 0 & 0 \\ 0 & -0.1061 & 0 \\ 0 & 0 & 0.2486 \end{pmatrix}.$$

Spin Hamiltonian parameters *D* and *E* (see Table 1) are obtained from the traceless ZFS tensor: $D = 3D_{zz}/2 = 0.373\ cm^{-1}$; $E = (D_{xx} - D_{yy})/2 = -0.018\ cm^{-1}$; $E/D = -0.049$.

The ^{13}C hf tensor as calculated by Orca (in MHz):

$$\hat{A} = \begin{pmatrix} 51.0378 & -0.3069 & -0.8427 \\ -0.3069 & 94.9545 & 0.1074 \\ -0.8427 & 0.1074 & -12.4163 \end{pmatrix}.$$

The hf tensor can then be transformed into the frame where the ZFS tensor is diagonal according to $\hat{A}' = R_D^T\, \hat{A}\, R_D$. The resulting matrix was used for the ^{13}C ENDOR simulations shown in Figure S13 B.

Diagonalization of the $\hat{A}$ matrix yields the hf eigenvalues given in Table 1:

$$\hat{A}_{diag} = \begin{pmatrix} 51.0 & 0 & 0 \\ 0 & 95.0 & 0 \\ 0 & 0 & -12.4 \end{pmatrix},\ R_A = \begin{pmatrix} -0.9999 & 0.0070 & 0.0133 \\ -0.0070 & -0.99997 & -0.0010 \\ 0.0133 & -0.0011 & 0.9999 \end{pmatrix}.$$

The order of hf eigenvalues (and the corresponding R_A columns) was chosen according to the labeling scheme used for the experimentally determined hf tensor values.

A rotation matrix from the ZFS frame to the hf frame can be constructed as $R_{\mathrm{ZFS}\to\mathrm{hf}} = R_A^T R_D$, where R_D represents the rotation from the ZFS frame to the molecular frame, and R_A^T represents the rotation from the molecular frame to the hf frame. The *eulang* function of EasySpin yields the corresponding Euler angles in the *z y*' *z*'' convention: [74.3, 1.3, 107.3]°, which correspond approximately to a 180° rotation around the *z* axis of the ZFS frame. The negative signs of the first two diagonal elements of R_A (together with the positive signs of the corresponding elements of R_D) reflect this ~180° rotation. Equivalently, the *x* and *y* axes of the hf frame can be inverted, in which case $R_{\mathrm{ZFS}\to\mathrm{hf}}$ will be additionally multiplied on the left by [-1 0 0; 0 -1 0; 0 0 1]. Then the Euler angles calculated by *eulang* are [74.3, 1.3, 287.3]° or, equivalently, [−105.7, −1.3, 107.3]° – the same set as calculated by Orca. Thus, the ^{13}C hf tensor frame is rotated with respect to the ZFS tensor axes by the Euler angles [−105.7, −1.3, 107.3]° in the *z y*' *z*'' notation, i.e., the noncollinearity of the two tensors is within 2° and is thus negligible.

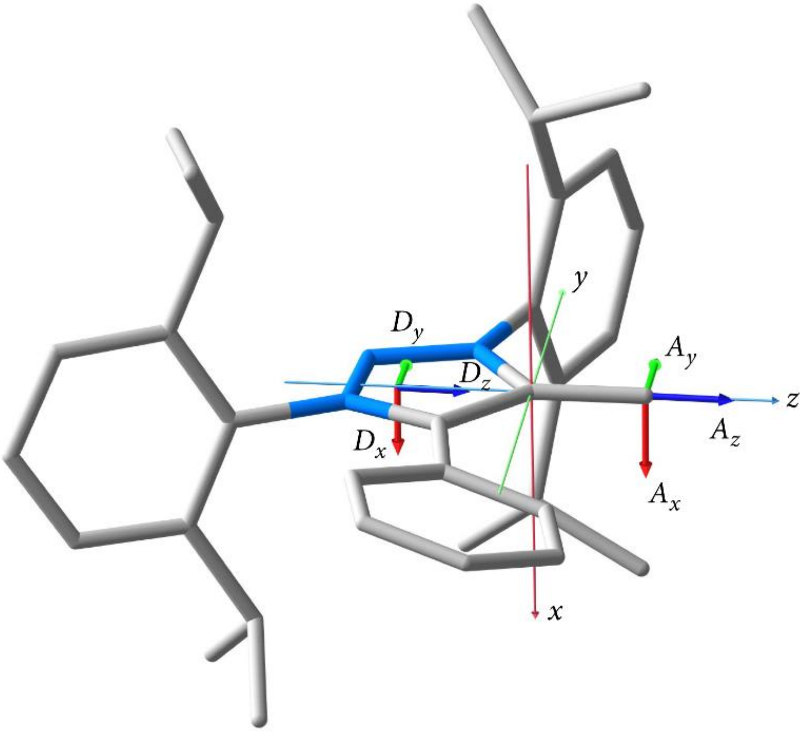

Figure S12. Molecular frame overlaid with the ZFS and ^{13}C hf tensor orientations calculated with Orca.

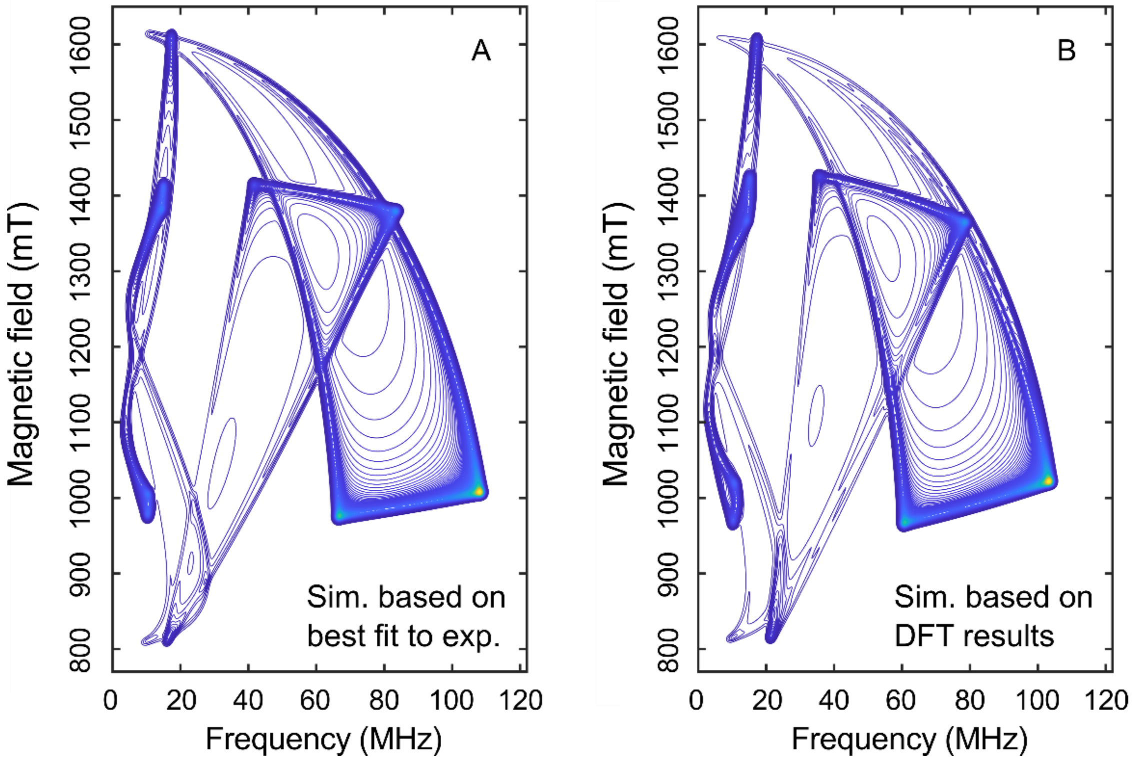

Figure S13. Simulated ^{13}C ENDOR patterns obtained over the entire EPR envelope using spin Hamiltonian parameters derived from (A) the best fit to the experimental ENDOR data at canonical field positions and (B) DFT calculations performed using Orca (see Table 1 and the description above).

4.8. Comparison of the vinylidene and carbene spin Hamiltonian parameters

Table S1. Experimental magnetic parameters of triplet ground state carbenes.

Compound	Solvent	T /K	$\|D\|$ /cm^{-1}	$\|E\|$ /cm^{-1}	$A(^{13}C)$ /MHz			$a_{iso}(^{13}C)$ /MHz	$T(^{13}C)$ /MHz			Ref.
Methylene 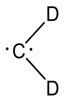	Xe	4	0.7525	n.a.	282	281	177	246.7	35.3	34.3	−69.7	33
	Xe	4	0.760[a]	0.0043[a]	275	280	196	250	24.7	29.7	−54.3	34
	Xe	4	0.7519[a]	0.0046[a]	274[b]	271[b]	171[b]	238.7	35.3	32.3	−67.7	35
Phenylmethylene 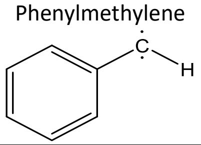	p-dichloro benzene	77	0.5150	0.0251	252	235	150	212.3	39.7	22.7	−62.3	36
Diphenylmethylene (diphenylcarbene) 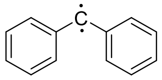	Benzophenone	77	0.4055	0.0194	214	196	115	175	39	21	−60	36
	Benzophenone (SC)	77	0.40505	0.01918	214.8	189.6	115.4	173.3	41.5	16.3	−57.8	37
Fluorenylidene 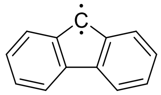	Fluorenone	77	0.4078	0.0283	308	278	202	262.7	45.3	15.3	−60.7	36
	Diazofluorene (SC)	77	0.40923	0.02828	307.3	280.0	203.4	263.6	43.8	16.4	−60.2	37
Vinylidene **2**	Toluene	6	0.377	0.0106	57.1	100.0	−7.2	50.0	7.1	50.0	−57.2	This work

[a]Values for $^{13}CD_2$. [b]Obtained at the X-band frequency.

4.9. Stability of the triplet vinylidene

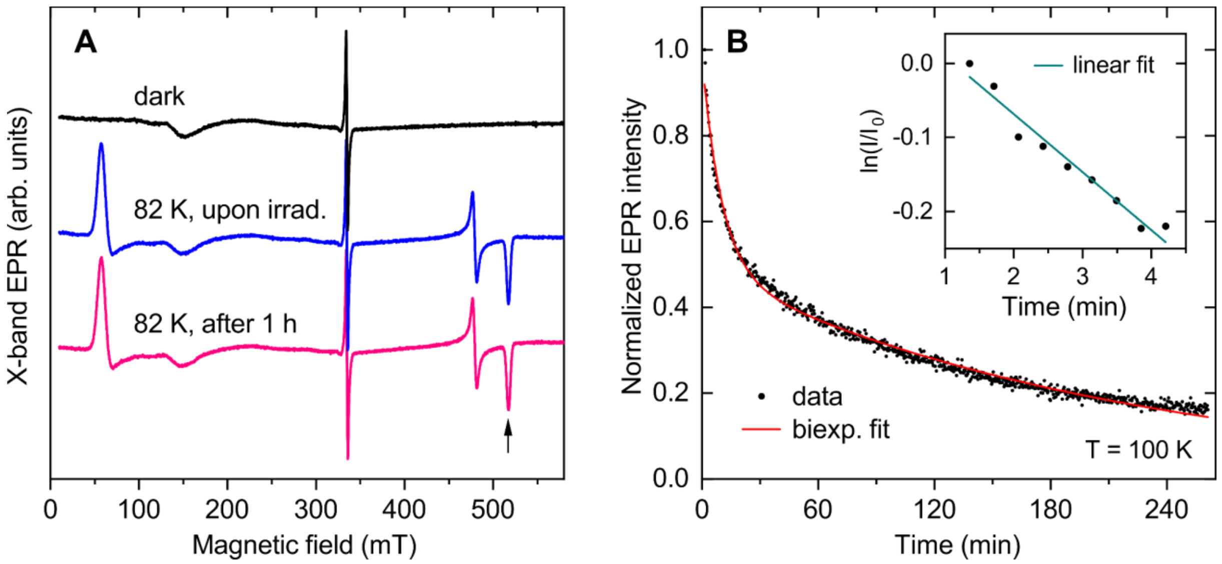

Figure S14. (A) CW X-band ($f_{MW} \approx 9.5$ GHz) EPR spectra of **2** in toluene collected at 82 K before irradiation (black trace), upon irradiation with UV light (λ = 395 nm, blue trace), and after 1 h at 82 K (pink trace). (B) Decay of the triplet species at 100 K monitored via intensity of the spectral feature marked with an arrow. Biexponential decay function $y = a_1 \cdot e^{-xk_1} + a_2 \cdot e^{-xk_2}$ was used to fit the experimental data; the best fit was achieved with $k_1 = 1.64(3) \cdot 10^{-3}$ s^{-1}, $k_2 = 7.79(6) \cdot 10^{-5}$ s^{-1}. The inset shows a plot of ln(I/I_0) vs time for the initial 20% of the vinylidene signal decay, following the approach described in literature.[38] A linear simulation yielded an initial rate constant of $k_{init} = 1.3(1) \cdot 10^{-3}$ s^{-1}.

5. References

1. Antoni, P. W.; Reitz, J.; Hansmann, M. M. N2/CO Exchange at a Vinylidene Carbon Center: Stable Alkylidene Ketenes and Alkylidene Thioketenes from 1,2,3-Triazole Derived Diazoalkenes. *J. Am. Chem. Soc.* **2021**, *143*, 12878-12885.
2. Neese, F.; Wennmohs, F.; Becker, U.; Riplinger, C. The ORCA quantum chemistry program package. *J. Chem. Phys.* **2020**, *152*, 224108.
3. Becke, A. D. Density-Functional Thermochemistry. III. The Role Of Exact Exchange. *J. Chem. Phys.* **1993**, *98*, 5648-5652.
4. Lee, C.; Yang, W.; Parr, R. G. Development of the Colle-Salvetti Correlation-Energy Formula Into a Functional of the Electron-Density. *Phys. Rev. B* **1988**, *37*, 785-789.
5. Weigend, F.; Ahlrichs, R. Balanced Basis Sets of Split Valence, Triple Zeta Valence and Quadruple Zeta Valence Quality for H to Rn: Design and Assessment of Accuracy. *Phys. Chem. Chem. Phys.* **2005**, *7*, 3297-3305.
6. Weigend, F. Accurate Coulomb-Fitting Basis Sets for H to Rn. *Phys. Chem. Chem. Phys.* **2006**, *8*, 1057-1065.
7. Neese, F.; Wennmohs, F.; Hansen, A.; Becker, U. Efficient, Approximate and Parallel Hartree–Fock and Hybrid DFT Calculations. A ‘Chain-of-Spheres’ Algorithm for the Hartree–Fock Exchange. *Chem. Phys.* **2009**, *356*, 98-109.
8. Staroverov, V. N.; Scuseria, G. E.; Tao, J.; Perdew, J. P. Comparative Assessment of a New Nonempirical Density Functional: Molecules and Hydrogen-Bonded Complexes. *J. Chem. Phys.* **2003**, *119*, 12129-12137.
9. Kossmann, S.; Kirchner, B.; Neese, F. Performance of Modern Density Functional Theory for the Prediction of Hyperfine Structure: Meta-GGA and Double Hybrid Functionals. *Mol. Phys.* **2007**, *105*, 2049-2071.
10. Pantazis, D. A.; Orio, M.; Petrenko, T.; Zein, S.; Bill, E.; Lubitz, W.; Messinger, J.; Neese, F. A New Quantum Chemical Approach to the Magnetic Properties of Oligonuclear Transition-Metal Complexes: Application to a Model for the Tetranuclear Manganese Cluster of Photosystem II. *Chem. Eur. J.* **2009**, *15*, 5108-5123.
11. Lohmiller, T.; Krewald, V.; Pérez Navarro, M.; Retegan, M.; Rapatskiy, L.; Nowaczyk, M. M.; Boussac, A.; Neese, F.; Lubitz, W.; Pantazis, D. A.; Cox, N. Structure, ligands and substrate coordination of the oxygen-evolving complex of photosystem II in the S_2 state: a combined EPR and DFT study. *Phys. Chem. Chem. Phys.* **2014**, *16*, 11877-11892.
12. Pantazis, D. A. First-Principles Calculation of Transition Metal Hyperfine Coupling Constants with the Strongly Constrained and Appropriately Normed (SCAN) Density Functional and its Hybrid Variants. *Magnetochemistry* **2019**, *5*, 69.
13. Orio, M.; Pantazis, D. A. Successes, challenges, and opportunities for quantum chemistry in understanding metalloenzymes for solar fuels research. *Chem. Commun.* **2021**, *57*, 3952-3974.
14. Gómez-Piñeiro, R. J.; Pantazis, D. A.; Orio, M. Comparison of Density Functional and Correlated Wave Function Methods for the Prediction of Cu(II) Hyperfine Coupling Constants. *ChemPhysChem* **2020**, *21*, 2667-2679.
15. Barone, V., Structure, Magnetic Properties and Reactivities of Open-Shell Species From Density Functional and Self-Consistent Hybrid Methods. In *Recent Advances in Density Functional Methods*, Chong, D. P., Ed. World Scientific: 1995; Vol. 1, pp 287-334.
16. Jakobsen, P.; Jensen, F. Probing basis set requirements for calculating hyperfine coupling constants. *J. Chem. Phys.* **2019**, *151*, 174107.

17. Sinnecker, S.; Neese, F. Spin-Spin Contributions to the Zero-Field Splitting Tensor in Organic Triplets, Carbenes and BiradicalsA Density Functional and Ab Initio Study. *J. Phys. Chem. A* **2006**, *110*, 12267-12275.
18. Riplinger, C.; Kao, J. P. Y.; Rosen, G. M.; Kathirvelu, V.; Eaton, G. R.; Eaton, S. S.; Kutateladze, A.; Neese, F. Interaction of Radical Pairs Through-Bond and Through-Space: Scope and Limitations of the Point-Dipole Approximation in Electron Paramagnetic Resonance Spectroscopy. *J. Am. Chem. Soc.* **2009**, *131*, 10092-10106.
19. Neese, F. Calculation of the zero-field splitting tensor on the basis of hybrid density functional and Hartree-Fock theory. *J. Chem. Phys.* **2007**, *127*, 164112.
20. Hess, B. A.; Marian, C. M.; Wahlgren, U.; Gropen, O. A mean-field spin-orbit method applicable to correlated wavefunctions. *Chem. Phys. Lett.* **1996**, *251*, 365-371.
21. Berning, A.; Schweizer, M.; Werner, H. J.; Knowles, P. J.; Palmieri, P. Spin-orbit matrix elements for internally contracted multireference configuration interaction wavefunctions *Mol. Phys.* **2000**, *98*, 1823-1833.
22. Neese, F. Efficient and Accurate Approximations to the Molecular Spin-Orbit Coupling Operator and Their Use in Molecular g-Tensor Calculations. *J. Chem. Phys.* **2005**, *122*, 034107.
23. Riplinger, C.; Neese, F. An efficient and near linear scaling pair natural orbital based local coupled cluster method. *J. Chem. Phys.* **2013**, *138*, 034106.
24. Riplinger, C.; Pinski, P.; Becker, U.; Valeev, E. F.; Neese, F. Sparse maps—A systematic infrastructure for reduced-scaling electronic structure methods. II. Linear scaling domain based pair natural orbital coupled cluster theory. *J. Chem. Phys.* **2016**, *144*, 024109.
25. Saitow, M.; Becker, U.; Riplinger, C.; Valeev, E. F.; Neese, F. A new near-linear scaling, efficient and accurate, open-shell domain-based local pair natural orbital coupled cluster singles and doubles theory. *J. Chem. Phys.* **2017**, *146*, 164105.
26. Ghafarian Shirazi, R.; Neese, F.; Pantazis, D. A. Accurate Spin-State Energetics for Aryl Carbenes. *J. Chem. Theory Comput.* **2018**, *14*, 4733-4746.
27. Ghafarian Shirazi, R.; Neese, F.; Pantazis, D. A.; Bistoni, G. Physical Nature of Differential Spin-State Stabilization of Carbenes by Hydrogen and Halogen Bonding: A Domain-Based Pair Natural Orbital Coupled Cluster Study. *J. Phys. Chem. A* **2019**, *123*, 5081-5090.
28. Ghafarian Shirazi, R.; Pantazis, D. A.; Neese, F. Performance of density functional theory and orbital-optimised second-order perturbation theory methods for geometries and singlet–triplet state splittings of aryl-carbenes. *Mol. Phys.* **2020**, *118*, e1764644.
29. Guo, Y.; Riplinger, C.; Becker, U.; Liakos, D. G.; Minenkov, Y.; Cavallo, L.; Neese, F. Communication: An improved linear scaling perturbative triples correction for the domain based local pair-natural orbital based singles and doubles coupled cluster method [DLPNO-CCSD(T)]. *J. Chem. Phys.* **2018**, *148*, 011101.
30. Angeli, C.; Cimiraglia, R.; Evangelisti, S.; Leininger, T.; Malrieu, J. P. Introduction of N-Electron Valence States for Multireference Perturbation Theory. *J. Chem. Phys.* **2001**, *114*, 10252-10264.
31. Angeli, C.; Cimiraglia, R.; Malrieu, J.-P. N-Electron Valence State Perturbation Theory: A Fast Implementation of the Strongly Contracted Variant. *Chem. Phys. Lett.* **2001**, *350*, 297-305.
32. Stoll, S.; Schweiger, A. EasySpin, a comprehensive software package for spectral simulation and analysis in EPR. *J. Magn. Reson.* **2006**, *178*, 42-55.

33. Wasserman, E.; Kuck, V.; Hutton, R.; Anderson, E.; Yager, W. 13C Hyperfine Interactions and Geometry of Methylene. *J. Chem. Phys.* **1971**, *54*, 4120-4121.
34. Bernheim, R.; Bernard, H.; Wang, P.; Wood, L.; Skell, P. 13C Hyperfine Interaction in CD2. *J. Chem. Phys.* **1971**, *54*, 3223-3225.
35. Bernheim, R. A.; Adl, T.; Bernard, H. W.; Songco, A.; Wang, P. S.; Wang, R.; Wood, L. S.; Skell, P. S. An electron paramagnetic resonance study of methylene. *J. Chem. Phys.* **1976**, *64*, 2747-2755.
36. Wasserman, E.; Trozzolo, A. M.; Yager, W. A.; Murray, R. W. ESR Hyperfine of Randomly Oriented Triplets: Structure of Substituted Methylenes. *J. Chem. Phys.* **1964**, *40*, 2408-2410.
37. Brandon, R. W.; Closs, G. L.; Davoust, C. E.; Hutchison, C. A.; Kohler, B. E.; Silbey, R. Electron Paramagnetic Resonance Spectra of the Ground - State Triplet Diphenylmethylene and Fluorenylidene Molecules in Single Crystals. *J. Chem. Phys.* **1965**, *43*, 2006-2016.
38. Platz, M. S., The Chemistry, Kinetics, and Mechanisms of Triplet Carbene Processes in Low-Temperature Glasses and Solids. In *Kinetics and Spectroscopy of Carbenes and Biradicals*, Platz, M. S., Ed. Springer US: Boston, MA, 1990; pp 143-211.
